# Time-series transcriptome comparison reveals the gene regulation network under salt stress in soybean (*Glycine max*) roots

**DOI:** 10.1186/s12870-022-03541-9

**Published:** 2022-03-31

**Authors:** Junmei Hu, Yongbin Zhuang, Xianchong Li, Xiaoming Li, Chanchan Sun, Zhaojun Ding, Ran Xu, Dajian Zhang

**Affiliations:** 1 College of Agronomy, State Key Laboratory of Crop Biology, Shandong Agricultural University, Tai’an, 271018 Shandong China; 2 Crop Research Institute, Shandong Academy of Agricultural Sciences, Ji’nan, 250131 Shandong China; 3 College of Life Sciences, Yantai University, Yan’tai, 264005 Shandong China; 4 The Key Laboratory of Plant Development and Environmental Adaptation Biology, Ministry of Education, School of Life Sciences, Shandong University, Qing’dao, 266237 Shandong China

**Keywords:** Soybean, RNA-seq, Root transcriptome, Salt stress

## Abstract

**Background:**

Soil salinity is a primary factor limiting soybean (*Glycine max*) productivity. Breeding soybean for tolerance to high salt conditions is therefore critical for increasing yield. To explore the molecular mechanism of soybean responses to salt stress, we performed a comparative transcriptome time-series analysis of root samples collected from two soybean cultivars with contrasting salt sensitivity.

**Results:**

The salt-tolerant cultivar ‘Qi Huang No.34’ (QH34) showed more differential expression of genes than the salt-sensitive cultivar ‘Dong Nong No.50’ (DN50). We identified 17,477 genes responsive to salt stress, of which 6644 exhibited distinct expression differences between the two soybean cultivars. We constructed the corresponding co-expression network and performed Gene Ontology term and Kyoto Encyclopedia of Genes and Genomes pathway enrichment analysis. The results suggested that phytohormone signaling, oxidoreduction, phenylpropanoid biosynthesis, the mitogen-activated protein kinase pathway and ribosome metabolism may play crucial roles in response to salt stress.

**Conclusions:**

Our comparative analysis offers a comprehensive understanding of the genes involved in responding to salt stress and maintaining cell homeostasis in soybean. The regulatory gene networks constructed here also provide valuable molecular resources for future functional studies and breeding of soybean with improved tolerance to salinity.

**Supplementary Information:**

The online version contains supplementary material available at 10.1186/s12870-022-03541-9.

## Background

Crops experience various abiotic stresses during their growth. Sodium (Na^+^) promotes plant growth at low concentrations but can be detrimental in high salinity conditions. Of the current 230 million hectares (ha) of irrigated land, 45 million ha (19.5%) are estimated to be affected by high salt; similarly, 32 million ha (2.1%) of the 1500 million ha used for dryland agriculture are affected by varying degrees of salt stress [1]. Affected areas are expected to increase by 10% annually due to changing global climatic conditions, low precipitation, high surface evaporation, weathering of native rocks, land clearing, irrigation with saline water, and poor cultural practices, reaching 50% of the world’s arable land by 2050 [2, 3]. When subjected to salt stress, plants experience osmotic stress, ionic toxicity and complex secondary effects [4]. High Na^+^ concentration in the soil leads to high osmotic pressure of the soil, disrupts cellular ion homeostasis, and prevents water and nutrient uptake from the soil, thus affecting plant growth and reducing yield [5, 6]. In addition, salt stress is accompanied by the accumulation of reactive oxygen species (ROS), which act as a secondary stress by causing the peroxidation of membrane lipids and the destruction of cellular membrane structures and proteins. Plants have accordingly developed multiple tolerance mechanisms to cope with salt stress, including adjustment and maintenance of ion homeostasis in response to osmotic stress, restoration of osmotic balance, and other metabolic and structural adaptations [6, 7].

Soybean (*Glycine max* (L.) Merr.), which originated in China and was domesticated ~ 6000–9000 years ago, is a vital source of both protein and cooking oil, providing 59% of the world’s oilseed production and 69% of the daily vegetable protein consumed [8]. Salt stress affects almost all aspects of soybean growth and development: germination, vegetative and reproductive growth, nodulation, leaf size, plant height, root length, shoot and root dry weight, seed size and weight [3, 7, 9]. Genes associated with tolerance to salt stress might be used in breeding new soybean varieties with high salt tolerance. Several genes participate in salt responses in soybean. These genes include ion regulator genes such as *HIGH-AFFINITY K*^*+*^ *TRANSPORTER 1;1* (*GmHKT1;1*), *GmHKT1;4*, *Arabidopsis K*^*+*^ *TRANSPORTER 1* (*GmAKT1*), *CATION DIFFUSION FACILITATOR 1* (*GmCDF1*), *qNaCl3* (*GmNcl*), *Na*^*+*^*/H*^*+*^ *ANTIPORTER 1* (*GmNHX1*), *GmNHX5*, *SALT OVERLY SENSITIVE 1* (*GmSOS1*) and *CHLORIDE CHANNEL 1* (*GmCLC1*), which play an important role in maintaining ion homeostasis under salt stress by regulating the transport and accumulation of Na^+^, potassium (K^+^), chloride (Cl^−^), and other ions [10–16]. Protein kinase genes such as Ser/Thr *PROTEIN KINASE 4* (*GmPKS4*), *NIMA-RELATED KINASE 1* (*GmNEK1*) and *CALCIUM-DEPENDENT PROTEIN KINASE 3* (*GmCDPK3*) [17–19], as well as transcription factor genes such as *GmNAC*, *GmWRKY*, *basic leucine-zipper* (*GmbZIP*), *HEAT-SHOCK FACTOR* (*GmHSF*), *GmMYB*, *PLANT HOMEODOMAIN* (*GmPHD*), *GmDREB* and *NUCLEAR FACTOR Y SUBUNIT A* (*GmNFYA*) [20–26] also respond to salt stress. Besides, phytohormones, including auxins, gibberellins, cytokinins, abscisic acid, ethylene, salicylic acid, jasmonates, brassinosteroids and strigolactones, also play major roles in mediating plant salt stress [6, 27]. However, few genes have been demonstrated to affect soybean yields in saline field conditions; little is known about salt signaling as soybean responds and adapts to high salinity. This lack of knowledge has greatly inhibited attempts to enhance salt tolerance in soybean.

Comparative differential gene expression analysis between contrasting genotypes is helpful to identify candidate genes and underlying molecular mechanisms. In this study, we selected the salt-tolerant soybean cultivar ‘Qi Huang No.34’ (QH34), a high-protein inbred line with high yields whose genome shows abundant genetic diversity inherited from six Chinese soybean accessions [28], and the salt-sensitive soybean cultivar ‘Dong Nong No.50’ (DN50), which was bred from the Canadian small soybean variety ‘Electron’ and is characterized by a short growth period, short plants, and single pods with many seeds. Here, we used transcriptome sequencing (RNA-seq) to identify salt stress-responsive genes in soybean. The identification of candidate genes and their associated mechanisms will provide a new basis for breeding salt-tolerant and high-yielding soybean varieties and contribute to increasing soybean productivity.

## Results

### Characteristics of QH34 and DN50 under salt stress

**Fig. 1:**
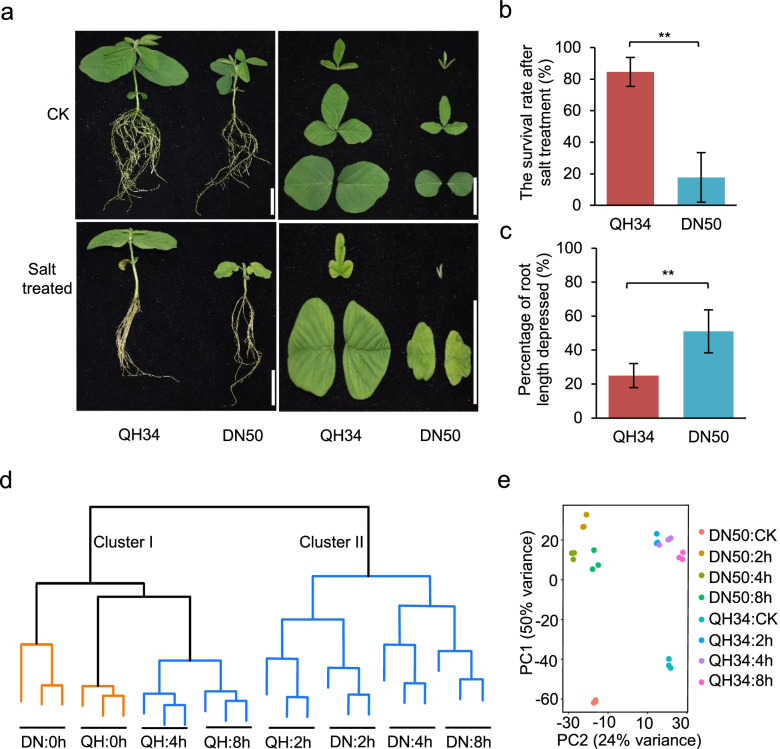
Salt tolerance in the two soybean cultivars DN50 and QH34 and RNA-seq analysis. **a**, Phenotypic differences between QH34 and DN50 in normal and salt-treated growth conditions. Bars = 5 cm. **b**, Survival rate of QH34 and DN50 plants after salt treatment. **c**, Percentage of root elongation repression by salt treatment. **d**, Hierarchical clustering of RNA-seq samples. e, PCA of RNA-seq samples

We evaluated the salt sensitivity of 22 soybean accessions (Table S1). Their phenotypes are shown in Fig. S1, in which Fig. S1a shows the phenotypes before salt treatment, Fig. S1b and S1c show the phenotypes grown in half-strength Hoagland’s solution without or with added NaCl, respectively. The growth of all 22 accessions was inhibited upon salt exposure (Fig. S1), but cultivar QH34 exhibited superior salt tolerance compared to the other accessions, while DN50 showed the lowest survival rate (Fig. 1a, b; Fig. S1). After 6 d of salt treatment, the leaves of QH34 remained green, whereas most leaves from DN50 turned yellow or died (Fig. 1a; Fig. S1). When plants were removed from salt stress and returned to half-strength Hoagland’s solution for 3 d, 84.5% of QH34 plants survived, whereas only 17.7% of DN50 plants survived (Fig. 1b). Root growth was also substantially slower under salt stress for both accessions, although the effect of salt stress was more severe for DN50 than for QH34, with the elongation of salt-treated roots repressed by 51.3% for DN50, but only 25.4% for QH34, relative to non-treated control roots (Fig. 1c). We therefore selected QH34 as a salt-tolerant accession and DN50 as a salt-hypersensitive accession for comparative root transcriptome analysis.

### RNA-seq data processing

We sequenced 24 root RNA libraries with an average number of 25.5 million 150-bp paired-end reads, ranging from 20.7 million to 30.6 million (Table 1). The mean rate of reads passing quality control was 99.3%, with an average GC content of 43.0% and duplicates of 43.3% (Fig. S2a; Table 1). We observed no significant difference in the sequencing output of the 24 samples, when estimated from Log_2_-normalized counts per million (Fig. S2b). We mapped the resulting clean reads to the soybean William 82 av2 reference genome using tophat2, reaching mapping rates of 94.1–96.0%, among which the percentage of unique mapped reads varied from 89.1 to 93.0% (Table 1), indicating that the RNA-seq libraries are of high quality.

**Table 1:** Evaluation of RNA-seq data from the two soybean cultivars DN50 and QH34

Sample Name	Toral reads (million)	Reads passed QC	% Dups	% GC	Overall mapping rate	Unique mapped rate
QH34:CK rep1	25.6	99.90%	47.20%	44%	94.30%	90.50%
QH34:CK rep2	22.1	99.60%	48.20%	43%	96.00%	91.50%
QH34:CK rep3	30.6	99.20%	40.90%	43%	95.20%	91.30%
QH34:2 h rep1	29.0	99.50%	48.80%	43%	95.60%	91.40%
QH34:2 h rep2	27.1	99.00%	43.70%	43%	94.30%	91.60%
QH34:2 h rep3	27.3	98.90%	48.40%	43%	94.50%	91.80%
QH34:4 h rep1	23.3	99.30%	32.70%	44%	94.30%	92.20%
QH34:4 h rep2	22.1	99.10%	29.30%	44%	94.50%	89.70%
QH34:4 h rep3	24.6	99.80%	32.20%	44%	94.30%	89.10%
QH34:8 h rep1	23.1	99.60%	29.70%	44%	95.30%	89.70%
QH34:8 h rep2	22.2	99.50%	31.90%	43%	95.00%	93.00%
QH34:8 h rep3	22.1	98.90%	29.30%	44%	94.90%	90.90%
DN50:CK rep1	29.7	99.10%	60.40%	43%	94.10%	90.30%
DN50:CK rep2	28.0	99.40%	48.40%	42%	95.20%	89.20%
DN50:CK rep3	20.7	99.40%	39.20%	42%	94.60%	91.40%
DN50:2 h rep1	26.0	98.90%	49.70%	43%	94.90%	91.40%
DN50:2 h rep2	28.1	98.80%	50.00%	43%	94.60%	91.70%
DN50:2 h rep3	23.9	99.80%	52.50%	42%	94.60%	89.20%
DN50:4 h rep1	25.8	99.40%	49.10%	43%	95.10%	90.70%
DN50:4 h rep2	28.3	99.10%	48.90%	43%	94.20%	90.70%
DN50:4 h rep3	22.0	98.80%	43.30%	43%	94.30%	92.20%
DN50:8 h rep1	29.1	99.10%	41.70%	43%	94.40%	92.70%
DN50:8 h rep2	23.6	98.90%	46.10%	43%	94.30%	89.50%
DN50:8 h rep3	27.6	99.30%	47.20%	43%	94.50%	89.90%
Average	25.5	99.26%	43.28%	43%	94.71%	90.90%

*rep* Repeat, *QC* Quality control, *Dups* Duplicates

Before proceeding with the identification of differentially expressed genes (DEGs), we performed hierarchical clustering (Fig. 1d) and principal component analysis (PCA; Fig. 1e) to estimate the similarity between samples. As shown in Fig. 1d, biological replicates globally clustered together. We identified two distinct clusters: cluster I with QH34 samples subjected to salt stress for 4 h and 8 h, as well as control samples for both QH34 and DN50, and cluster II with all other salt-treated samples. Root samples exposed to salt for 4 h and 8 h appeared to be more similar to the corresponding control samples for QH34, indicating that QH34 is less affected by salt treatment, in agreement with the strong salt tolerance observed in this variety. By contrast, samples collected from QH34 after only 2 h of salt treatment grouped with all salt-treated DN50 samples. PCA also indicated a good reproducibility across the biological samples, as they each defined a single group per sample type. In addition, the two major principal components can well separate two materials as well as the same material with difference treatment (Fig. 1e). In summary, both hierarchical clustering and PCA reflected the good quality and repeatability of our samples.

### Identification of DEGs

**Fig. 2:**
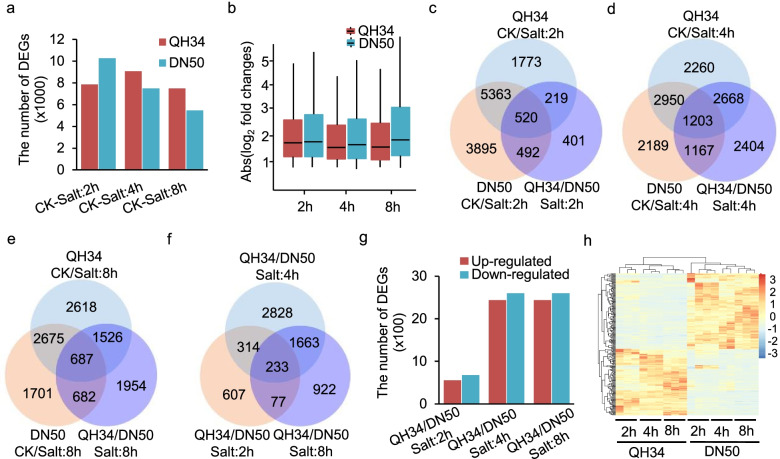
Time-dependent comparisons of DEGs between QH34 and DN50. **a**, Number of salt-responsive genes identified at each time point. **b**, Boxplot showing the average changes of DEGs at each time point, shown as absolute Log_2_(fold change). **c**–**e**, Venn diagrams showing the extent of overlap between DEGs from QH34 and DN50 after 2 h (c), 4 h (d), or 8 h (e) of salt treatment. **f**, Venn diagram showing shared and unique DEGs across the three time points. **g**, Identification of upregulated and downregulated genes **h**, Heatmap showing the expression patterns of DEGs across the three salt treatment durations

We proceeded to identify DEGs in two steps. First, we determined the salt-responsive genes by comparing salt-treated samples with the controls for each cultivar at three time points (2 h, 4 h and 8 h). We detected fewer DEGs for QH34 than for DN50 after 2 h of salt treatment, while the opposite was true after 4 h and 8 h (Fig. 2a). We identified 13,890 unique salt-responsive genes in QH34, compared to 12,098 unique salt-responsive genes in DN50 across all three time points. We also obtained lower average fold-change values for the DEGs in QH34 than for those in DN50 (Fig. 2b) across all time points, indicating that when the salt stress is perceived, more genes were responsive in QH34, but the fluctuation of the expression of genes was controlled at a smaller scale compared to DN50. Second, we identified DEGs between the two cultivars for each time point (Fig. 2c–e). Genes that showed significant differences in expression between the two cultivars but were not salt-responsive were not analyzed further, yielding a final list of 1231 DEGs after 2 h of salt treatments, 5038 DEGs after 4 h, and 2895 DEGs after 8 h. The smaller number of DEGs identified after 2 h suggested that similar gene networks are triggered by salt stress in the two cultivars at this early time point. Of the 7875 salt-responsive genes in QH34 and 10,270 salt-responsive genes in DN50 after 2 h of exposure to salt, only 1231 genes were differentially expressed between the cultivars, with 520 (42.2%) were shared by QH34 and DN50, while the remaining 492 (40.0%) and 219 (17.8%, Fig. 2c) were DN50- and QH34-specific DEGs, respectively. By contrast, the relative proportion of shared DEGs identified after 4 h and 8 h of salt treatment was lower than after 2 h, as 1203 of 5038 DEGs (23.9%) and 687 of 2895 DEGs (23.7%) were shared for the 4 h and 8 h salt treatments, respectively, suggestive of cultivar-specific responses to salt stress occurred (Fig. 2d, e). We also compared DEGs across the three time points and identified 233 genes showing significant expression differences between QH34 and DN50 at all three time points, which defined universal salt-responsive genes (uDEGs). Other DEGs, either specific to a single time point or shared by two time points, were considered as specific salt-responsive genes (sDEGs) (Fig. 2f). We obtained similar numbers of upregulated and downregulated genes after salt stress (Fig. 2g). The 233 uDEGs showed distinct expression patterns between QH34 and DN50 (Fig. 2f), most exhibiting consistent behavior across all time points (Fig. 2h).

### Classification of the expression patterns of the DEGs

**Fig. 3:**
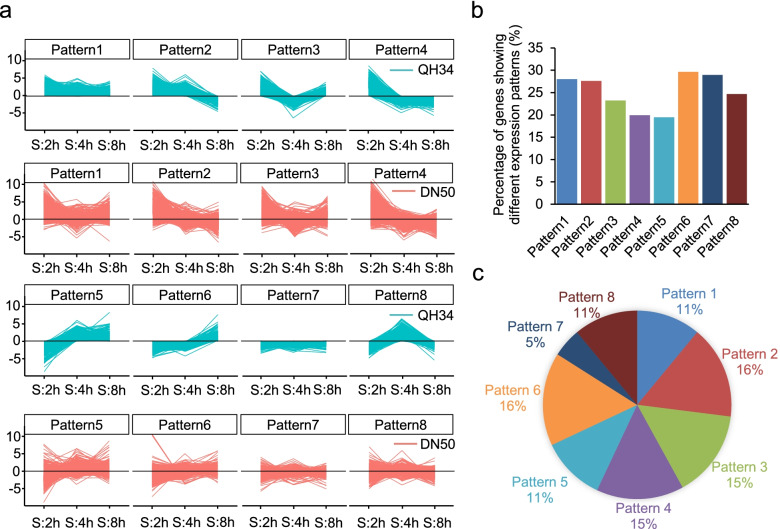
Analysis of DEGs expression patterns. **a**, Using the expression patterns of DEGs in QH34 as reference (cyan), the expression of the corresponding genes in DN50 were plotted (red). **b**, Percentage of genes showing different expression patterns between QH34 and DN50. **c**, Distribution of each expression pattern type

We classified the expression patterns of the DEGs into eight categories by calculating their transcript abundance relative either to the control or to the previous time point (Methods), using QH34 as a reference. Overall, we observed similar expression patterns for DEGs between QH34 and DN50 (Fig. 3a). However, within each of the eight defined categories, an average of 25.2% of DEGs displayed a different expression pattern from that of its cohort, ranging from 19.5% (pattern 5) to 29.6% (pattern 6; Fig. 3b). Expression patterns 2 and 6 were present in the most DEGs, each accounting for 16% (Fig. 3c). Expression patterns of 2 and 6 were opposite to each other as well as patterns 3 and 8: expression of genes belonging to pattern 2 was upregulated at 2 h and 4 h and downregulated at 8 h, while expression of genes belonging to pattern 6 was first downregulated at 2 h and 4 h and then upregulated at 8 h after treatment. Genes belongs to pattern 3 were upregulated at 2 h and 8 h and downregulated at 4 h after salt treatment, while expression of genes belonging to pattern 8 were downregulated at 2 h and 8 h and upregulated at 4 h in QH34 (Fig. 3a). By further dissecting the expression pattern of DEGs belong to those four major groups showing different expression patterns between QH34 and DN50, we found, the majority of the genes were either upregulated or downregulated across all three time points in both cultivars (Fig. 3a, b). Comparing to the continuously up- or down-regulating the expression of a gene, the expression patterns 2,3, 6 and 8 are likely to maintain cell homeostasis. DEGs with differential expression between QH34 and DN50 are good candidates for a gene regulatory network and a starting point for studying the molecular mechanism contributing to salt tolerance in QH34.

### Construction of gene network, gene ontology (GO) and Kyoto encyclopedia of genes and genomes (KEGG) enrichment analysis

**Fig. 4:**
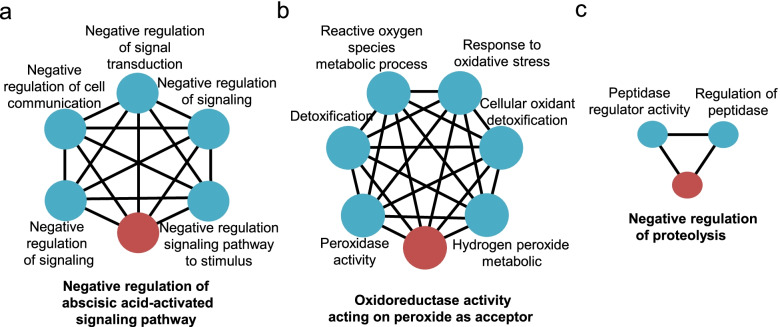
GO enrichment analysis for uDEGs. **a**, Negative regulation of ABA-activated signaling. **b**, Oxidoreductase activity signaling. **c**, Negative regulation of proteolysis signaling

To facilitate interpreting the biological functions of the DEGs identified above and understand the molecular mechanism controlling salt tolerance in soybean, we clustered DEGs based on the Pearson’s correlation coefficient values calculated from their expressions and built undirected networks based on their patterns of co-expression for uDEGs and sDEGs independently. Of 233 uDEGs, 203 (87.1%) successfully clustered into three groups, containing 95 (40.8%), 83 (35.6%) and 25 (10.7%) DEGs each. For cluster I, the most enriched GO term was associated with the negative regulation of abscisic acid (ABA) signaling (Fig. 4a). Examination of the expression pattern of the key genes within this pathway, such as *Glyma.02G250200*, *Glyma.11G222600*, *Glyma.02G302400*, *Glyma.13G090600* and *Glyma.18G035000*, we found that compared to the controls, the expression levels of those genes were both upregulation in QH34 and DN50, particularly at 2 h, although the induction of gene expression was much more modest in QH34 than in DN50 (Fig. S3). Since these genes are negative regulators of ABA signaling, we hypothesize that ABA levels may be lower in DN50 upon salt stress compared to QH34. The most enriched GO term in Cluster II was associated with oxidoreductase activity, responses to oxidative stress (Fig. 4b). The expression of this set of genes was higher in QH34 than in DN50 for control samples (Fig. S4). However, the induction of gene expression by salt stress was more pronounced in QH34 compared to DN50, such as observed for *Glyma.16G055900*. For genes whose expression was repressed by exposure to salt, the repression was weaker in QH34 compared to DN50, as with *Glyma.13G306900* and *Glyma.12G195600*. Conversely, for genes whose expression was lower in QH34 than DN50 in the control samples, the decline in gene expression was less marked in QH34 than in DN50, such as observed for *Glyma.08G321100* (Fig. S4). Cluster III mainly comprised genes involved in the negative regulation of proteolysis (Fig. 4c; S5). Other GO terms enriched for uDEGs were involved in cellular ion homeostasis, signal transduction, protein modification and carbohydrate metabolism, etc. (Fig. S6). KEGG enrichment of uDEGs indicated that six pathways are significantly enriched: glycolysis, phenylpropanoid biosynthesis, glutathione metabolism, carbon fixation in photosynthetic organism, plant hormone signal transduction and mitogen-activated protein kinase (MAPK) signaling pathway (Fig. S7).

**Fig. 5:**
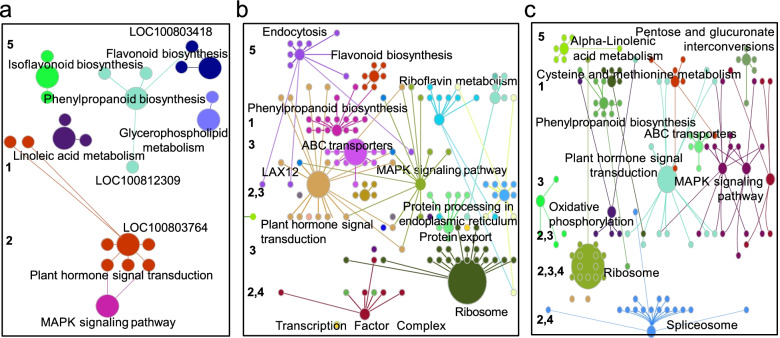
KEGG pathway analysis for sDEGs. **a**, **b** and **c** show pathway analysis at 2 h, 4 h and 8 h, respectively. 1, extracellular; 2, intracellular; 3, plasma membrane; 4, nucleus; 5, undefined

We also performed a functional characterization of sDEGs at 2 h, 4 h and 8 h after treatment, resulting in gene networks with three, four and four clusters, respectively (Fig. S8). As shown in Fig. 5, we identified pathways specific to each time point as well as pathways shared across all three time points. Phenylpropanoid biosynthesis, plant hormone signal transduction and MAPK signaling pathway were shared by all three time points, although the underlying genes varied across the time points. As previously mentioned, we identified only 1231 DEGs at 2 h, and the most significant pathway enriched at this time point was plant hormone signal transduction, followed by MAPK signaling pathway (Fig. 5a). Ribosome metabolism became the predominant pathway at both 4 h and 8 h (Fig. 5b, c), prompting us to examine the expression pattern of genes involved in the most enriched pathway at each time point between QH34 and DN50 (Fig. S9, 10, 11 and 12). Of the genes involved in plant hormone signaling, we detected 20 (43.5%) responsive to auxin stimulus that appear to encode four auxin/indole-3-acetic acid (IAA) family proteins based on sequence similarity (Fig. S9). Genes similar to *Arabidopsis IAA4* (*Glyma*.*19G161000, Glyma.02G000500*) and *IAA26* (*Glyma*.*07G015200*) were upregulated upon salt stress in DN50, while *IAA27* (*Glyma*.*08G203100*) was downregulated in QH34 under the same conditions. By contrast, a gene with similarity to *AUXIN-RESPONSE FACTOR 5* (*ARF5*; *Glyma.14G217700*) was significantly induced in QH34 upon salt stress, while *ARF9* (*Glyma*.*07G134800*) was strongly downregulated in DN50. Taken together, these results indicate that auxin contributes positively to salt tolerance in soybean during the tested period. Genes specifically involved in gibberellic acid (GA; *Glyma*.*14G086600*, *Glyma*.*11G155100*), salicylic acid (SA; *Glyma.15G232000*), ethylene (ET; *Glyma*.*08G105000*), jasmonic acid (JA; *Glyma*.*10G031900*, *Glyma*.*14G086600*) and brassinosteroids (BRs; *Glyma.13G224300*) were also more highly expressed in QH34 than in DN50 (Fig. S9). The expression of 73.9% (34 of 46) of genes involved in MAPK signaling pathway was induced by salt stress, with higher levels in QH34 than in DN50 for 80.4% (37 of 46; Fig. S10). Examination of genes involved in phenylpropanoid biosynthesis revealed genes encoding caffeic acid O-methyltransferase (CMOT; *Glyma*.*06G295700*, *Glyma*.*12G109800*, *Glyma*.*20G003500*) as being highly induced at 4 h and 8 h in the salt-tolerant cultivar QH34 (Fig. S11). We also observed significant expression differences for 133 genes involved in ribosome metabolism at 4 h and 8 h, as they were expressed at lower levels in the salt-tolerant cultivar QH34 than in DN50 (Fig. S12).

### Functional validation of selected DEGs

**Fig. 6:**
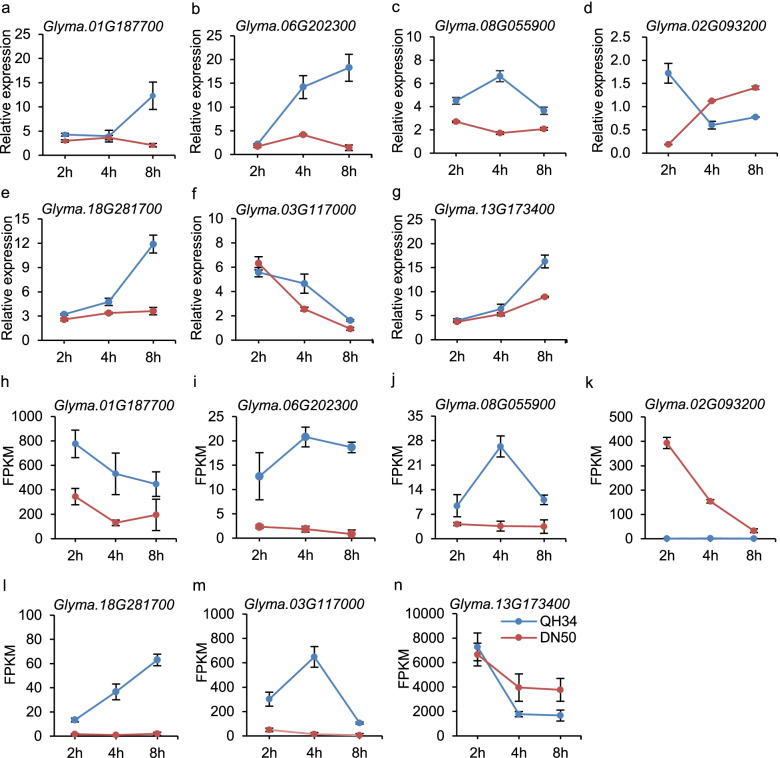
Validated DEGs by RT-qPCR. **a**–**g**, Relative expression levels of DEGs *Glyma.01G187700* (**a**), *Glyma.06G202300* (**b**), *Glyma.08G055900* (**c**), *Glyma.02G093200* (**d**), *Glyma.18G281700* (**e**), *Glyma.03G117000* (**f**) and *Glyma.13G173400* (**g**), as determined by RT-qPCR. h–n, FPKM values for DEGs *Glyma.01G187700* (**h**), *Glyma.06G202300* (**i**), *Glyma.08G055900* (**j**), *Glyma.02G093200* (**k**), *Glyma.18G281700* (**l**), *Glyma.03G117000* (**m**) and *Glyma.13G173400* (**n**)

**Fig. 7:**
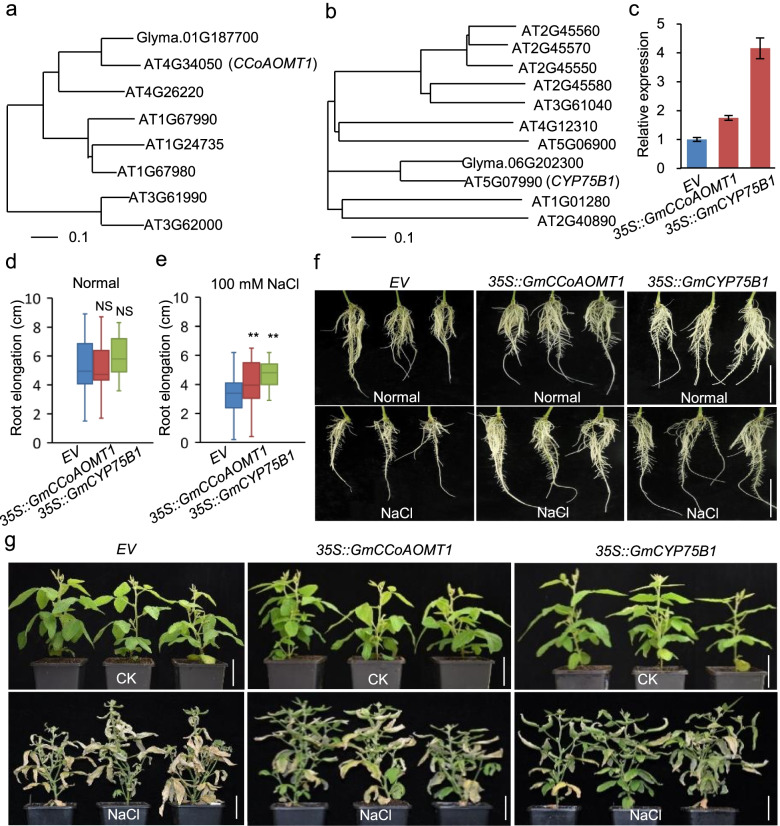
Phylogenetic analysis and functional validation of *GmCCoAOMT1* and *GmCYP75B1*. **a** and **b**, Phylogenetic analysis of Glyma.01G187700 and Glyma.06G202300 from soybean and Arabidopsis orthologous proteins, respectively. **c**, Relative expression of *Glyma.01G187700* and *Glyma.06G202300* in transgenic soybean hairy roots, as determined by RT-qPCR. **d** and **e**, Hairy root elongation between empty vector (*EV*) and overexpression (*OE*) plants in normal and high salt conditions. **f**, Phenotypes of hairy roots between *EV* and *OE* in normal and high salt conditions. **g**, Phenotypes of above-ground parts of *EV* and *OE* plants before (upper) and after (lower panel) salt treatment. NS, not significant. **p < 0.01. Bar = 5 cm

We validated by Real Time Quantitative PCR (RT-qPCR) the expression patterns of selected seven DEGs (Fig. 6; Table 2). Five genes (*Glyma.06G202300*, *Glyma.01G187700*, *Glyma.08G055900*, *Glyma.03G117000* and *Glyma.18G281700*) showed basically consistent results by RNA-seq and RT-qPCR. We selected two of them (*Glyma.06G202300* and *Glyma.01G187700*) with high expression levels in QH34 for functional validation using the soybean hairy root system in the salt-sensitive cultivar DN50 (Fig. 7a, b). A phylogenetic analysis of Glyma.01G187700 and orthologous *Arabidopsis* proteins (Fig. 7a) revealed that *Glyma.01G187700* encodes caffeoyl-CoA O-methyltransferase 1 (*GmCCoAOMT1*). Under salt stress, *Glyma.01G187700* was more highly expressed in QH34 than in DH50 (Fig. 6a, h). Overexpression of *Glyma.01G187700* conferred higher tolerance to salt than the empty vector control, as evidenced by the greater root elongation observed in the presence of 100 mM NaCl but not in the absence of salt stress (Fig. 7c–f). We also characterized above-ground phenotypes of the transgenic plants. When treated with 200 mM NaCl for 12 d, the above-ground parts of *OE* plants also exhibited higher vitality than empty vector controls, as most leaves remained green, in sharp contrast to the yellowing or dying leaves of the controls (Fig. 7g).

**Table 2:** *OE* and RT-qPCR primers used for functional validation of DEGs

Primer name		Primer sequence
RT qPCR	Actin_F	CGGTGGTTCTATCTTGGCATC
	Actin_R	GTCTTTCGCTTCAATAACCCTA
	Glema.01G187700_F	CCCTATGGAATGGGTCCGTG
	Glyma.01G187700_R	ATCCCATCACCAACGGGAAG
	Glyma.06G202300_F	GATGGCAATGGAGGGTGTGA
	Glyma.06G202300_R	CCTATCTCGACCCACAACGG
	Glyma.08G055900_F	ATCTGGTCTTGCTCCGGTTG
	Glyma.08G055900_R	GGGTGGATCTGGCCTCTTTC
	Glyma.18G281700_F	TTGCGGGAGATAGTTCCGAC
	Glyma.18G281700_R	ATACACGACCACCAATTCTGGG
	Glyma.03G117000_F	GGAATGAGCCCAAGGGTGTT
	Glyma.03G117000_R	TGCACTTGCGACTGAAGAAGA
	Glyma.13G173400_F	TGCCCTGGAGTCAATCTGG
	Glyma.13G173400_R	CCATGCTAACTTTGGCGTCAC
	Glyma.02G093200_F	AGATGGTCCAGGAAGCAGAAAAA
	Glyma.02G093200_R	TGGTTTCCATCCAACCACTGA
OE	Glyma.01G187700_F	gagaacacgggggactctagaATGACTGTCATTAAGGAAGAGCAACA
	Glyma.01G187700_R	ggactgaccacccggggatccGATGATGCGGCGGCACAG
	Glyma.06G202300_F	gagaacacgggggactctagaATGTCTCCATTGATTGTTGCCTT
	Glyma.06G202300_R	ggactgaccacccggggatccGGAAGACATTGAGTACACATGTGGTG

*Glyma.06G202300* encoded a member of the cytochrome P450 superfamily (*GmCYP75B1*), likely involved in oxidation–reduction process (Fig. 7b). The expression of *Glyma.06G202300* was induced by salt stress and continuously increased at 4 h and 8 h after treatment in both QH34 and DN50, though with a lower amplitude in DN50 (Fig. 6b, i; S4). Overexpression of *Glyma.06G202300* in transgenic soybean hairy roots increased its transcript levels over four-fold relative to the empty vector control, as measured by RT-qPCR (Fig. 7c). Root elongation were slightly, but not significantly, longer than empty vector controls when *Glyma.06G202300 OE* hairy roots were grown in control conditions consisting of half-strength Hoagland’s solution (Fig. 7d). After salt stress exposure (100 mM NaCl) for 3 d, root elongation is significantly inhibited, and the *OE* of *Glyma.06G202300* was able to reduce the inhibition of root elongation caused by the salt stress (Fig. 7e, f). Likewise, the above-ground part of *Glyma.06G202300 OE* plants exhibited higher vitality compared to the empty vector controls after being treated with 200 mM NaCl for 12 d, with most leaves remaining green (Fig. 7g).

## Discussion

Plant responses to salt stress are complex, as evidenced by the > 17,000 genes whose expression changed under salt stress over the short time period examined in the present study. Crosstalk between pathways often further complicates the dissection of the underlying molecular mechanism. Here, we performed GO term and KEGG enrichment analysis on clustered gene co-expression networks and focused on identifying pathways with the greatest differences between the salt-tolerant cultivar QH34 and the salt-sensitive cultivar DN50. This analysis revealed several candidate pathways with a role in salt responses, as detailed below.

### Oxidoreduction

Oxidoreduction reactions involve the transfer of electrons between donor and acceptor molecules and are essential for basic life functions including photosynthesis and respiration. Peroxidase can detoxify H_2_O_2_ and respond to environmental stresses such as wounding, pathogen attacks and oxidative stress [29]. Here, the main pathway identified for uDEGs Cluster II was oxidoreductase activity (Fig. 4b). The genes in this cluster were more highly expressed in the salt-tolerant cultivar QH34 than in the salt-sensitive cultivar DN50, indicating their positive role in the salt tolerance of soybean (Fig. S4).

### MAPK

MAPKs are activated by various biotic and abiotic stresses. Salt stress induces two well-characterized MAPKs activating signaling molecules, phosphatidic acid and ROS via nicotinamide adenine dinucleotide phosphate (NADPH)-oxidase [30]. *Glycine max* MAP kinase 1 (GMK1) is regulated by phosphatidic acid and H_2_O_2_ during salt stress [31]. The three most differentially expressed genes within the MAPK signaling pathway were *MAPKK2* (*Mitogen-activated protein kinase kinase 2*), *BAK1* (*BRI1-associated receptor kinase 1*) and *RboH* (*Respiratory burst oxidase homolog*). All three genes were significantly upregulated in the salt-tolerant cultivar QH34 and slightly induced in the salt-sensitive cultivar DN50. In *Arabidopsis*, MAPKK2 contributes to cold and salt signaling by regulating MAPK6 and MAPK4 [32]. Overexpression of poplar (*Populus trichocarpa*) *MAPKK* enhances salt tolerance in tobacco (*Nicotiana tabacum*) [33]. BAK1 is the co-receptor of the membrane receptor BRASSINOSTEROID INSENSITIVE 1 (BRI1); under salt stress conditions, BAK1 works with BRI1 to transduce the BR signal to BR SIGNALING KINASE 1 (BSK1) and activate the phosphatase BRI1 SUPPRESSOR 1 (BSU1). BSU1 then inhibits the kinase BR-INSENSITIVE 2 (BIN2) and promotes the translocation of the transcription factors BRASSINAZOLE-RESISTANT 1 (BZR1)/BRI1 EMS SUPPRESSOR 1 (BES1) to the nucleus to induce the expression of BR-responsive genes, enhancing salt tolerance [34]. The *RboH* identified here is homologous to *AtrbohF*, which functions in maintaining cellular Na^+^/K^+^ homeostasis under salt stress, as a mutation in *AtrbohF* caused imbalance of Na^+^/K^+^ homeostasis, resulting in salt sensitivity [35]. In wheat (*Triticum aestivum*), high Na^+^ concentrations induce NADPH oxidase-dependent ROS generation, which elevates Ca^2+^ levels in roots [36]. The coordination of the MAPK pathway with plant hormones may play a key role in perceiving and transmitting external signals and maintain cellular homeostasis.

### Phenylpropanoid biosynthesis genes

Phenylpropanoids are a group of plant secondary metabolites derived from phenylalanine that play crucial roles in the plant life cycle. Phenylalanine is first converted to cinnamic acid by deamination, and the resulting product is hydroxylated and methylated to generate coumaric acid and other acids with a phenylpropane unit. Aldehydes and alcohols can be produced when the reduction of CoA-activated carboxyl groups occurs. The alcohols are called monolignols and are the building blocks for lignin, a major component of the cell wall. Phenylpropanoid biosynthesis is important under salt stress [37]. In the present study, 76.9% of DEGs involved in phenylpropanoid biosynthesis were induced by salt stress, while 84.6% of the same DEGs showed on average a higher expression level in the salt-tolerant cultivar QH34 under salt stress compared to DN50, suggesting their positive roles in salt tolerance. The most upregulated genes (*Glyma*.*06G295700*, *Glyma*.*12G109800*, *Glyma*.*20G003500*) were annotated as encoding CMOT, orthologs to *Arabidopsis ATOMT1* (*A*t*5*g*54160*)*.* The cell walls of *omt1* mutants were more susceptible to enzymatic hydrolysis and displayed a higher digestibility compared to the wild type [38]. In the case of maize (*Zea mays*), mutation in the gene also led to the improved digestibility and decreased lignin levels of the cell wall [39]. Transgenic *Arabidopsis* plants overexpressing *CMOT* from *Carex rigescens*)*,* a stress-tolerant grass, not only exhibited enhanced salt stress tolerance but also produced more lateral roots and accumulated more proline and chlorophyll contents than the wild type [40]. The high induction of *CMOT* expression observed in QH34 under salt stress may contribute to the outstanding salt tolerance of this cultivar.

### Plant hormones and salt tolerance

Plant hormones regulate plant growth and development, as well as responses to abiotic and biotic stresses [6, 27]. In the present study, we determined that the ABA as the most enriched plant hormone signaling pathway for uDEGs while auxin as the most significant pathway enriched in sDEGs. The roles of ABA in salt stress have been repeatedly reported. ABA is the most efficient plant hormone for lowering the concentrations of Na^+^ and Cl^−^ and the Na^+^/K^+^ ratio and for increasing K^+^ and Ca^2+^ concentrations, proline accumulation, and soluble sugar contents [41]. Hormonal priming with ABA (50 ppm) increases wheat capacity to cope with salinity [42]. Similarly, ABA-treated wheat plants exhibit higher salt tolerance and a significant decrease in Na^+^ concentrations in flag leaves [43]. A salt-tolerant variety of rice (*Oryza sativa*) displays a higher ability than a sensitive cultivar to produce ABA, underscoring the positive contribution of ABA to reducing stress effects [44]. In agreement, endogenous ABA levels increase in the rice root subjected to salt stress [45]. In soybean, ABA content in leaves similarly increases with exposure to elevated salt stress [46]. In the present study, we established that genes involved in negative regulation of ABA biosynthesis are upregulated in DN50, but only slightly upregulated or downregulated in QH34 (Fig. S3). We hypothesize that ABA content is higher in QH34 than in DN50, which may contribute to the greater salt tolerance of QH34.

Auxin, which plays major roles in plant growth, controls root growth and the proliferation of lateral roots. Local auxin minima serve as signals that trigger the transition from cell division to cell differentiation in the *Arabidopsis* root [47]. Upon salt stress, we observed that negative regulators of auxin signaling are either significant upregulated or downregulated to a lesser extent in the salt-sensitive cultivar DN50, while positive regulators of auxin signaling were mainly upregulated in the salt-tolerant cultivar QH34 (Fig. S9). The exogenous application of IAA enhances the ability of maize plants to respond to abiotic stress [48]. Although the role of auxin in salt stress is likely to be complex by controlling the balance between growth and stress response and crosstalk with other phytohormones, auxin positively contributed to the salt resistance observed in QH34 during the period examined here.

Other phytohormones such as GA, SA, JA, ET and BRs may also play important roles in adjusting plant growth to survive in high salt conditions, and complex crosstalk between phytohormones was anticipated. However, as genes related to these pathways were less enriched and showed no significant differences in expression between QH34 and DN50, they are not further discussed here.

### The role of ribosome metabolism in salt tolerance

Ribosome biogenesis is a central process in any cell. In rapidly growing cells, high levels of ribosomal proteins (RPs) are translated. Maintaining cell functions requires a tight coordination between ribosomal RNAs and RPs, and the alteration of any step along the ribosome assembly process may negatively influence cell growth and cause proteotoxic stress [49]. Our comparison of the root transcriptome of the salt-tolerant cultivar QH34 and the salt-sensitive cultivar DN50 indicated that most genes participating in ribosome assembly and metabolism are strongly repressed at 4 h and 8 h after salt treatment in QH34, and the expression depression of some essential structural constituent of ribosome, such as *Glyma.12G162300* (encoding 30S ribosomal protein S20), *Glyma.11G201700* (encoding 50S ribosomal protein L17) could be observed at as early as 2 h in QH34 (Fig. S12). In comparison, the same genes remained unchanged or slightly downregulated at 2 h in the salt-sensitive cultivar DN50. Our results suggest that lower ribosome activity may be crucial during the early stages of cell response to salt stress. In the common ice plant (*Mesembryanthemum crystallinum*), which can tolerate Na^+^ concentrations exceeding that found in sea water and complete its life cycle, the ribosome-inactivating protein gene *RIP1* is highly induced upon salt stress [50]. The capacity of the cell to mount a timely and appropriate response to stress is critical for determining cell fate. Typically, the stress responses first allow cells to adapt and recover from the specific stress; if the stimulatory insult cannot be resolved, cell death signaling pathways will be initiated [51]. The timely adjustment of ribosome activity observed in QH34 may help cells better adapt to salt stress or act as a signal for activating downstream pathways, as observed in a process referred to as the ribotoxic stress response [52].

## Conclusions

Our root transcriptome comparison analysis between the salt-tolerant cultivar QH34 and the salt-sensitive cultivar DN50 at three time points over the course of salt stress showed that more genes display an active response to salt stress in QH34 but with smaller amplitudes relative to DN50. We identified 17,477 unique salt stress responsive genes across three time points, of which 6644 genes differentiate QH34 and DN50 at least at one of the time points tested, suggesting that salt tolerance in soybean is complex. Further clustering, gene network construction and GO/KEGG enrichment analysis of DEGs suggested that during the time period observed, genes involved in plant hormone signaling, oxidoreduction, phenylpropanoid biosynthesis, MAPK signaling and ribosome metabolism may play crucial roles in response to salt stress. We also validated one of the DEGs, as evidenced by the observed salt tolerance in DN50 resulting from its overexpression. In summary, the findings reported here advance our understanding of the molecular mechanisms regulating salt tolerance in soybean. The identified genes will be useful in breeding new soybean varieties with improved salt tolerance.

## Methods

### Plant materials and application of salt stress

The salt-tolerant cultivar Qi Huang No.34 (QH34) and the salt-sensitive cultivar Dong Nong No.50 (DN50) were selected for root deep transcriptome sequencing. Plants were grown in a growth chamber under 16 h of light and 8 h of dark (28 °C/20 °C, 50% relative humidity). Four days after sowing (vermiculite: nutritional soil = 1:1), seedlings were transferred to hydroponic growth conditions in half-strength Hoagland’s solution (pH 6.0), which was renewed every 3 d until reaching the VC growth stage (unifoliolate leaves are fully developed and the first trifoliolate leaf appears). Twenty plants selected from each cultivar at similar growth status were either subjected to 150 mM NaCl in half-strength Hoagland’s solution (pH 6.0) or maintained in half-strength Hoagland’s solution without NaCl as the control for 6 d. The roots were cleaned to remove traces of NaCl and the plants were then returned to half-strength Hoagland’s solution for 3 d, at which point the survival rate was calculated. Test of significance (*p* ≤ 0.05) was performed in R using Student’s *t*-test.

### RNA extraction, library preparation and transcriptome sequencing

For each cultivar, root composite samples from ten plant were harvested 2 h, 4 h and 8 h after salt treatment or from the control plants (as described above), three biological replicates. Total RNA was extracted using the RNeasy Plant Mini Kit (QIAgene) according to the manufacturer’s instructions. The quality of RNA was evaluated on 1% (w/v) agarose gels; the integrity of the extracted RNA was then assessed using an Agilent Bioanalyzer 2100 system (Agilent Technologies) before library construction. High-quality RNA was used for RNA-seq library using the Truseq RNA Library Prep Kit (Illumina). RNA libraries were sequenced at Novogene (Beijing, China) to generate paired-end 150-bp reads on a HiSeq2000 platform.

### Data processing and identification of DEGs

Raw data in fastq format were first quality control using Trimmomatic (ILLUMINACLIP: Adapter_seq.fa:2:30:10) [53] to remove sequencing adaptors, low-quality bases (Phred score ≤ 20), and reads containing ploy-N. Quality-trimmed data were further evaluated using fastQC to calculate Q20, Q30 and GC contents of the clean data. For the identification of DEGs, the soybean (*Glycine max*) William 82 av2 reference genome and its corresponding annotation file were retrieved from soybase (https://www.soybase.org/). Genome index was built using Bowtie2 [54]. Paired-end reads having passed quality controls were aligned to the soybean reference genome using Tophat2 [55] with default parameters. The resulting bam files were used for reads duplication rate estimation with R package DupRadar. Median-normalized and Log_2_-transformed data was used for hierarchical clustering and principal component analysis to estimate the similarity between samples. FeatureCounts was used to count the number of reads mapping to each gene. Identification of DEGs between treatments was performed with the R package DESeq2 [56]. Genes showing at least two-fold expression changes (Log_2_(fold change) ≥1 or ≤ − 1) and with an adjusted *p* value ≤0.01 were considered as differentially expressed. To classify these DEGs and construct the gene network contributing to salt tolerance in QH34, salt-responsive genes were identified by comparing salt-treated samples with their corresponding controls for each cultivar. Genes showing significant expression changes between the control and the salt-treated plants were considered as salt-responsive genes. Only salt-responsive DEGs were further analyzed between QH34 and DN50 to identify genes contributing to salt tolerance in QH34.

### Analysis of DEGs expression patterns

The expression patterns of DEGs induced by salt stress in each cultivar were calculated separately. The expression changes for a gene were calculated according to the ratio of fragments per kb of exon model per million mapped fragments (FPKMs) for a gene after salt treatment (2 h, 4 h, or 8 h) relative to FPKMs for the matching control (CK) or relative to another salt treatment time that less it (2 h or 4 h). Depending on whether it is positive (upregulated) or negative (downregulated) relative to its expression at the preceding time point, it was classified into 8 types.

### Gene network construction, GO functional enrichment and KEGG pathway analysis

To construct gene co-expression networks and study the coordination of gene expression under salt stress, a raw gene count table generated from FeatureCounts was normalized by median normalization using the EBSeq R package [57], then Log_2_ transformation was applied to the normalized data and genes with low count were removed from downstream analysis (threshold was set based on the normalized reads counts< 10). Calculation of correlation coefficients was performed with the R package psych (method = “ Pearson ”). The resulting correlation tables were further filtered based on absolute correlation (> 0.9) and adjusted *p* value (< 0.01). Network statistics were calculated using the R package igraph [58]. The visualization and clustering gene network were performed using Cytoscape. Briefly, the calculated correlation table and the network statistics tables were imported into Cytoscape, then the loaded data was further analyzed using clusterMaker [59] (cluster method = “community clustering (Glay)”) with default parameters. The clustered gene network was visualized using Degree Sorted Circle Layout to identify the hub genes (the top most connected genes in each cluster). GO functional enrichment and KEGG [60] pathway analysis were conducted in ClueGO; gene IDs were converted to Ensembl gene IDs (*Glycine max* [3847]). For GO functional enrichment, we focused on Biological Process. For both GO and KEGG enrichment analysis, the minimum number of genes was set to five for a GO term to be significantly enriched, and groups with > 50% overlap were merged. GO term/pathway network connectivity (kappa score) was set to Medium (0.5). DEGs were classified into two groups: one group containing DEGs annotated as salt-responsive shared by both QH34 and DN50, and the other containing time point-specific genes for QH34. For both groups, GO function and KEGG pathway enrichment analysis were independently performed for each gene network cluster.

### RT-qPCR analysis

Total RNA was extracted using the Trizol reagent. The cDNA was synthesized following the instructions of the HiScript® II Q RT SuperMix for qPCR (+gDNA wiper) synthesis kit (Vazyme, Nanjing, China). qPCR was performed on a Step One Plus™ Real-Time PCR system (Applied Biosystems, USA) using ChamQ Universal SYBR qPCR Master Mix (Vazyme, Nanjing, China). Three independents biological replicates were analyzed. Samples used for RNA extraction were collected from plant roots treated as mentioned above.

### Overexpression vector construction and plant transformation

The seven genes were filtered for RT-qPCR validation based on their functional annotations, expression patterns, respective pathways and the data from literature reports [34, 61–64]. Two genes of them were filtered for functional validation, and the primers (Table 2) used for target gene amplification were designed on the Vazyme website (https://crm.vazyme.com/cetool/singlefragment.html) and the coding sequences (Williams 82) were amplified. The resulting PCR products were ligated into the 35S-pBI121 (kanamycin resistance) vector and transformed into *Escherichia coli* DH5α by freeze-thaw method. Plasmids with the correct insert and free of mutations were introduced into Agrobacterium (*Agrobacterium rhizogenes*) strain K599 (streptomycin-resistant) using the freeze-thaw method. K599 colonies with the target construct were used to inoculate 10 mL YEP liquid medium and incubated at 28 °C for 10 h with shaking (220 rpm). After 10 h, 100 μL bacterial culture was spread onto a YEP solid plate and cultured in an incubator (28 °C) for 2 d for subsequent hairy root experiments.

### Validation of gene function through soybean hairy root system

We generated transgenic hairy roots overexpressing the target gene (*OE*) and evaluated their salt sensitivity by growth in half-strength Hoagland’s solution with or without added NaCl, side by side with control hairy roots transformed with empty vector (*EV*). The salt-sensitive cultivar DN50 was used for functional validation by overexpressing the candidate genes. DN50 was sown in soil (a 1:1 mix of vermiculite and nutritional soil). Six days after sowing, the tip of a 1-mL medical syringe was used to puncture four wounds 0.5–1 cm below the cotyledons in a cross pattern. Then, cultures from the relevant Agrobacterium colonies were smeared onto the four wounds with pearl wool. The seedlings were covered with a clear plastic lid to maintain high humidity. Six days later, the plastic cover was removed and the infected area was covered with vermiculite to maintain humidity. Then, 14 d later, the taproot was cut off when the hairy root was ~ 5–10 cm in length and cultured in half-strength Hoagland’s solution (pH 6.0) for 3 d to adapt environment, and consistent seedlings were selected for salinity treatment of 3 d with 100 mM NaCl; controls received no NaCl. The root length before and after salt treatment was measured and the root relative elongation was calculated to evaluate salt tolerance. In addition, about 35 d after Agrobacterium infection, overexpressing hairy root plants and the control were transplanted into mixed soil. After 3 d, the hairy root plants were irrigated with half-strength Hoagland’s solution containing 200 mM NaCl, or half-strength Hoagland’s solution without NaCl as the control group. Each plant was irrigated with 50 mL solution every 2 d. After 12 d of salt treatment, the above-ground phenotypes were determined.

## Supplementary Information

**Additional file 1: Fig. S1.** The measurement of salt tolerance in 22 soybean accessions.

**Additional file 2: Fig. S2.** Evaluation of RNA-seq data from the two soybean cultivars DN50 and QH34.

**Additional file 3: Fig. S3.** Heatmap of negative regulation abscisic acid activity signaling pathway.

**Additional file 4: Fig. S4.** Heatmap of oxidoreductase activity signaling pathway.

**Additional file 5: Fig. S5.** Heatmap of negative regulation of proteolysis signaling pathway.

**Additional file 6: Fig. S6.** GO enrichment analysis for uDEGs.

**Additional file 7: Fig. S7.** KEGG pathway analysis of uDEGs.

**Additional file 8: Fig. S8.** Pathway analysis of gene network clusters for sDEGs.

**Additional file 9: Fig. S9.** Heatmap of hormone signaling pathways.

**Additional file 10: Fig. S10.** Heatmap of MAPK signaling pathway.

**Additional file 11: Fig. S11.** Heatmap of phenylpropanoid biosynthesis signaling pathway.

**Additional file 12: Fig. S12.** Heatmap of ribosome metabolism biosynthesis signaling pathway.

**Additional file 13: Table S1.** The source information of 22 soybean germplasms.

## Data Availability

The transcriptome data was submitted to NCBI (https://www.ncbi. nlm.nih.gov/geo/), and could be accessed using project ID/accession ID PRJNA766706. 22 soybean germplasm resources have been authorized.

## Abbreviations

DN50: Dong Nong No.50
QH34: Qi Huang No.34
DEGs: Differentially expressed genes
PCA: Principal component analysis
uDEGs: Universal salt-responsive genes
sDEGs: Specific salt-responsive genes
MAPK: Mitogen-activated protein kinase
ROS: Reactive oxygen species
NADPH: Nicotinamide adenine dinucleotide phosphate
RPs: Ribosomal proteins
ABA: Abscisic acid
MAPKK: Mitogen-activated protein kinase kinase
GA: Gibberellic acid
SA: Salicylic acid
ET: Ethylene
JA: Jasmonic acid
BRs: Brassinosteroids
BRI1: BR-insensitive 1
BAK1: BRI1-associated receptor kinase 1
RboH: Respiratory burst oxidase homolog
BSK1: BR signaling kinase 1
BSU1: BRI1 suppressor 1
CMOT: Caffeic acid O-methyltransferase
GO: Gene ontology
KEGG: Kyoto encyclopedia of genes and genomes
RNA-seq: Transcriptome sequencing
RT-qPCR: Real time quantitative PCR
OE: Overexpression
EV: Empty voter
FPKMs: Fragments per kb of exon model per million mapped fragments
