# Supplementary material for: Time-series transcriptome comparison reveals the gene regulation network under salt stress in soybean (Glycine max) roots
Source: BMC Plant Biol. 2022 Mar 31;22:157. doi: 10.1186/s12870-022-03541-9 (PMC8969339; doi:10.1186/s12870-022-03541-9)
Supplement: Supplementary file 1 — Additional file 1: Fig. S1. The measurement of salt tolerance in 22 soybean accessions. [file 12870_2022_3541_MOESM1_ESM.pptx]

## Slide 1
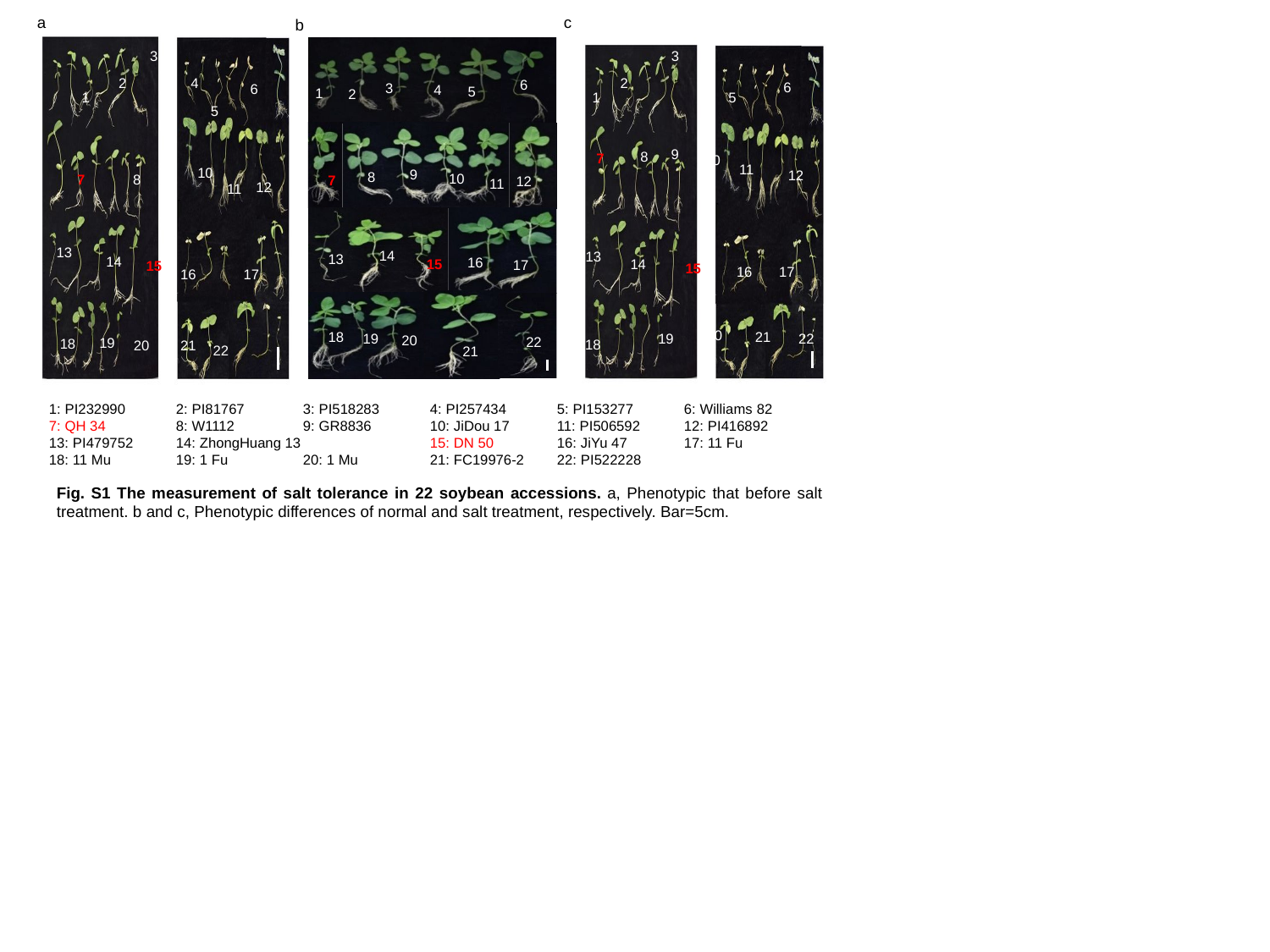

c
a
b
6
3
4
5
1
2
9
8
10
7
12
11
14
13
16
15
17
18
19
20
22
21
3
2
4
6
1
5
10
7
8
9
12
11
13
14
15
17
16
19
18
20
21
22
3
2
4
6
1
5
9
8
7
10
11
12
13
14
15
17
16
20
21
19
22
18
1: PI232990 	2: PI81767 	3: PI518283 	4: PI257434 	5: PI153277 	6: Williams 82
7: QH 34	8: W1112	9: GR8836	10: JiDou 17 	11: PI506592	12: PI416892
13: PI479752	14: ZhongHuang 13 	15: DN 50	16: JiYu 47 	17: 11 Fu
18: 11 Mu	19: 1 Fu	20: 1 Mu	21: FC19976-2	22: PI522228
Fig. S1 The measurement of salt tolerance in 22 soybean accessions. a, Phenotypic that before salt treatment. b and c, Phenotypic differences of normal and salt treatment, respectively. Bar=5cm.
