# Supplementary material for: Time-series transcriptome comparison reveals the gene regulation network under salt stress in soybean (Glycine max) roots
Source: BMC Plant Biol. 2022 Mar 31;22:157. doi: 10.1186/s12870-022-03541-9 (PMC8969339; doi:10.1186/s12870-022-03541-9)
Supplement: Supplementary file 2 — Additional file 2: Fig. S2. Evaluation of RNA-seq data from the two soybean cultivars DN50 and QH34. [file 12870_2022_3541_MOESM2_ESM.pptx]

## Slide 1
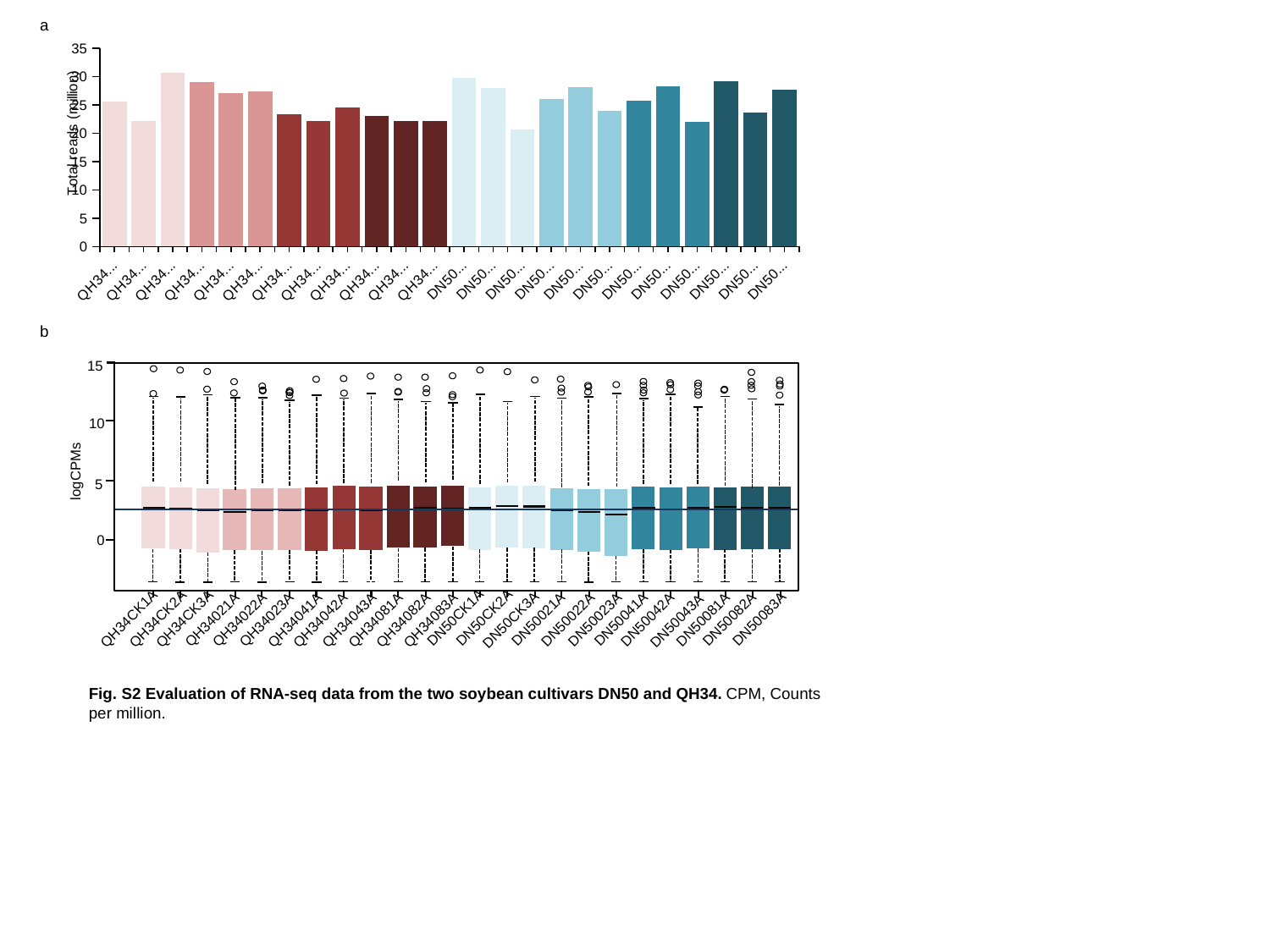

a
### Chart
| Category | Toral reads (million) |
|---|---|
| QH34CK1A | 25.6 |
| QH34CK2A | 22.1 |
| QH34CK3A | 30.6 |
| QH34021A | 29.0 |
| QH34022A | 27.1 |
| QH34023A | 27.3 |
| QH34041A | 23.3 |
| QH34042A | 22.1 |
| QH34043A | 24.6 |
| QH34081A | 23.1 |
| QH34082A | 22.2 |
| QH34083A | 22.1 |
| DN50CK1A | 29.7 |
| DN50CK2A | 28.0 |
| DN50CK3A | 20.7 |
| DN50021A | 26.0 |
| DN50022A | 28.1 |
| DN50023A | 23.9 |
| DN50041A | 25.8 |
| DN50042A | 28.3 |
| DN50043A | 22.0 |
| DN50081A | 29.1 |
| DN50082A | 23.6 |
| DN50083A | 27.6 |b
QH34CK1A
QH34CK2A
QH34CK3A
QH34021A
QH34022A
QH34023A
QH34041A
QH34042A
QH34043A
QH34081A
QH34082A
QH34083A
DN50CK1A
DN50CK2A
DN50CK3A
DN50021A
DN50022A
DN50023A
DN50041A
DN50042A
DN50043A
DN50081A
DN50082A
DN50083A
15
10
logCPMs
5
0
Fig. S2 Evaluation of RNA-seq data from the two soybean cultivars DN50 and QH34. CPM, Counts per million.
