# Supplementary material for: Time-series transcriptome comparison reveals the gene regulation network under salt stress in soybean (Glycine max) roots
Source: BMC Plant Biol. 2022 Mar 31;22:157. doi: 10.1186/s12870-022-03541-9 (PMC8969339; doi:10.1186/s12870-022-03541-9)
Supplement: Supplementary file 3 — Additional file 3: Fig. S3. Heatmap of negative regulation abscisic acid activity signaling pathway. [file 12870_2022_3541_MOESM3_ESM.pptx]

## Slide 1
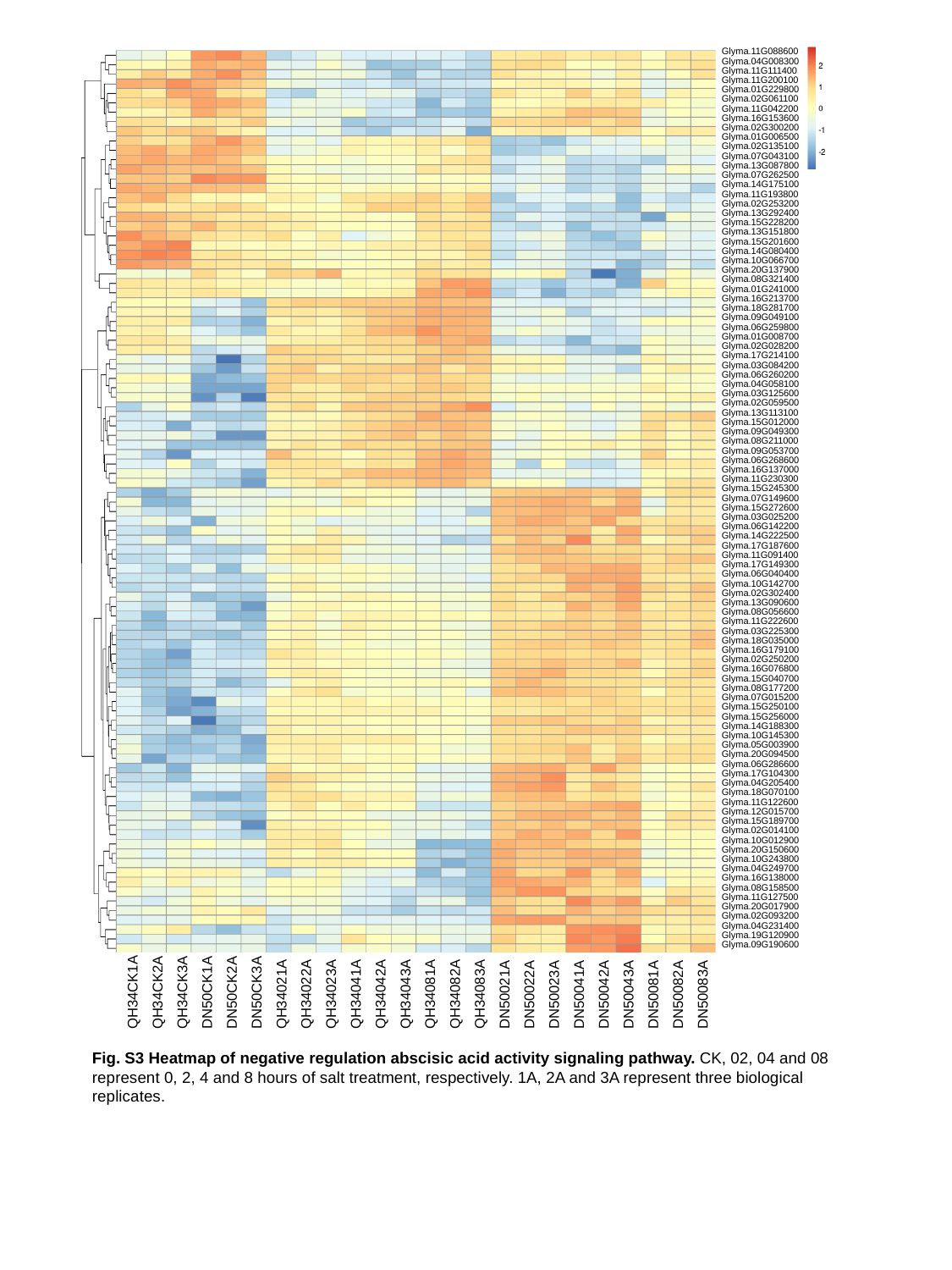

Glyma.11G088600
Glyma.04G008300
Glyma.11G111400
Glyma.11G200100
Glyma.01G229800
Glyma.02G061100
Glyma.11G042200
Glyma.16G153600
Glyma.02G300200
Glyma.01G006500
Glyma.02G135100
Glyma.07G043100
Glyma.13G087800
Glyma.07G262500
Glyma.14G175100
Glyma.11G193800
Glyma.02G253200
Glyma.13G292400
Glyma.15G228200
Glyma.13G151800
Glyma.15G201600
Glyma.14G080400
Glyma.10G066700
Glyma.20G137900
Glyma.08G321400
Glyma.01G241000
Glyma.16G213700
Glyma.18G281700
Glyma.09G049100
Glyma.06G259800
Glyma.01G008700
Glyma.02G028200
Glyma.17G214100
Glyma.03G084200
Glyma.06G260200
Glyma.04G058100
Glyma.03G125600
Glyma.02G059500
Glyma.13G113100
Glyma.15G012000
Glyma.09G049300
Glyma.08G211000
Glyma.09G053700
Glyma.06G268600
Glyma.16G137000
Glyma.11G230300
Glyma.15G245300
Glyma.07G149600
Glyma.15G272600
Glyma.03G025200
Glyma.06G142200
Glyma.14G222500
Glyma.17G187600
Glyma.11G091400
Glyma.17G149300
Glyma.06G040400
Glyma.10G142700
Glyma.02G302400
Glyma.13G090600
Glyma.08G056600
Glyma.11G222600
Glyma.03G225300
Glyma.18G035000
Glyma.16G179100
Glyma.02G250200
Glyma.16G076800
Glyma.15G040700
Glyma.08G177200
Glyma.07G015200
Glyma.15G250100
Glyma.15G256000
Glyma.14G188300
Glyma.10G145300
Glyma.05G003900
Glyma.20G094500
Glyma.06G286600
Glyma.17G104300
Glyma.04G205400
Glyma.18G070100
Glyma.11G122600
Glyma.12G015700
Glyma.15G189700
Glyma.02G014100
Glyma.10G012900
Glyma.20G150600
Glyma.10G243800
Glyma.04G249700
Glyma.16G138000
Glyma.08G158500
Glyma.11G127500
Glyma.20G017900
Glyma.02G093200
Glyma.04G231400
Glyma.19G120900
Glyma.09G190600
QH34CK1A
QH34CK2A
QH34CK3A
DN50CK1A
DN50CK2A
DN50CK3A
QH34021A
QH34022A
QH34023A
QH34041A
QH34042A
QH34043A
QH34081A
QH34082A
QH34083A
DN50021A
DN50022A
DN50023A
DN50041A
DN50042A
DN50043A
DN50081A
DN50082A
DN50083A
Fig. S3 Heatmap of negative regulation abscisic acid activity signaling pathway. CK, 02, 04 and 08 represent 0, 2, 4 and 8 hours of salt treatment, respectively. 1A, 2A and 3A represent three biological replicates.
