# Supplementary material for: Time-series transcriptome comparison reveals the gene regulation network under salt stress in soybean (Glycine max) roots
Source: BMC Plant Biol. 2022 Mar 31;22:157. doi: 10.1186/s12870-022-03541-9 (PMC8969339; doi:10.1186/s12870-022-03541-9)
Supplement: Supplementary file 4 — Additional file 4: Fig. S4. Heatmap of oxidoreductase activity signaling pathway. [file 12870_2022_3541_MOESM4_ESM.pptx]

## Slide 1
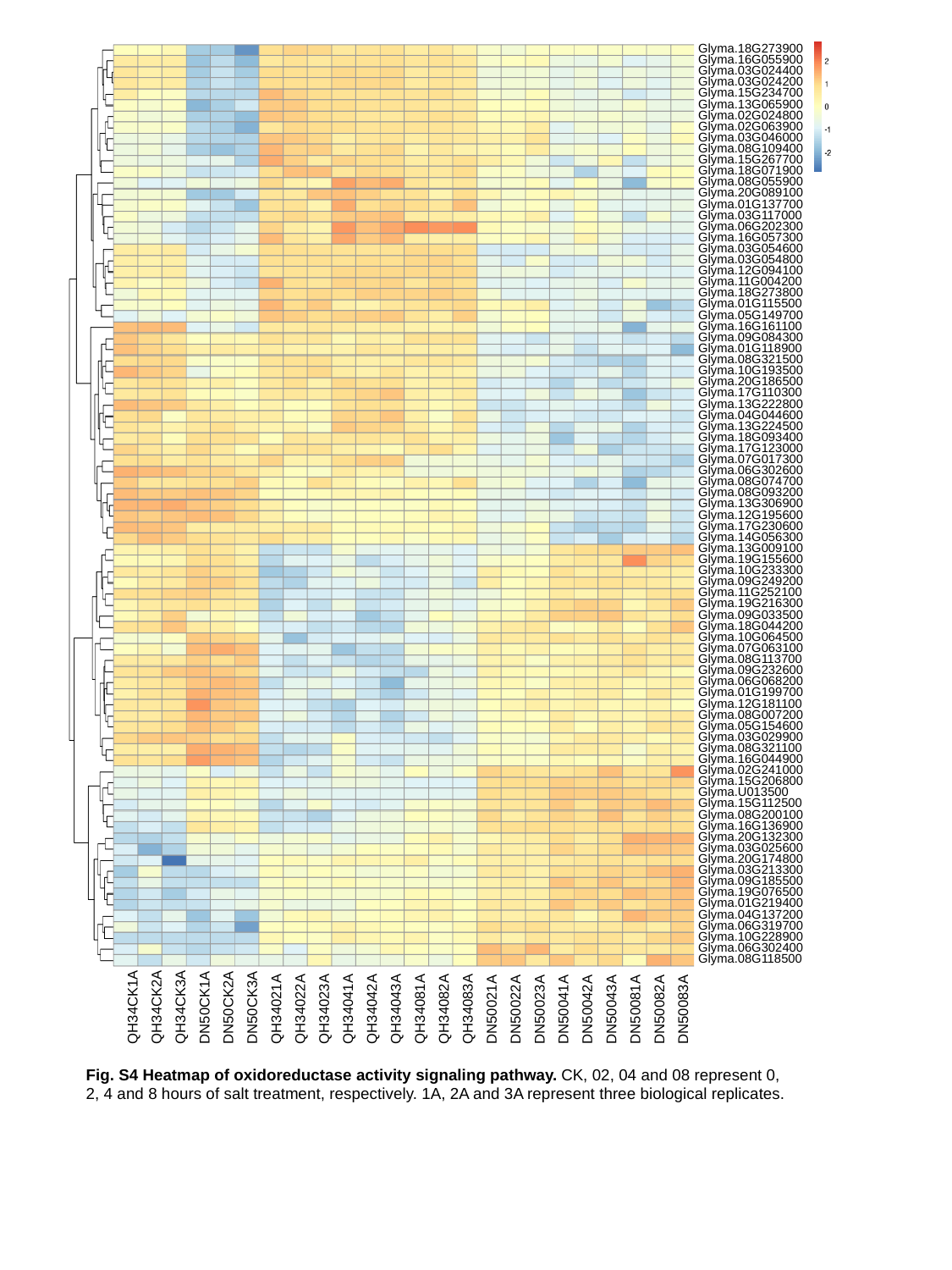

Glyma.18G273900 Glyma.16G055900 Glyma.03G024400 Glyma.03G024200 Glyma.15G234700 Glyma.13G065900 Glyma.02G024800 Glyma.02G063900 Glyma.03G046000 Glyma.08G109400 Glyma.15G267700 Glyma.18G071900 Glyma.08G055900 Glyma.20G089100 Glyma.01G137700 Glyma.03G117000 Glyma.06G202300 Glyma.16G057300 Glyma.03G054600 Glyma.03G054800 Glyma.12G094100 Glyma.11G004200 Glyma.18G273800 Glyma.01G115500 Glyma.05G149700 Glyma.16G161100 Glyma.09G084300 Glyma.01G118900 Glyma.08G321500 Glyma.10G193500 Glyma.20G186500 Glyma.17G110300 Glyma.13G222800 Glyma.04G044600 Glyma.13G224500 Glyma.18G093400 Glyma.17G123000 Glyma.07G017300 Glyma.06G302600 Glyma.08G074700 Glyma.08G093200 Glyma.13G306900 Glyma.12G195600 Glyma.17G230600 Glyma.14G056300 Glyma.13G009100 Glyma.19G155600 Glyma.10G233300 Glyma.09G249200 Glyma.11G252100 Glyma.19G216300 Glyma.09G033500 Glyma.18G044200 Glyma.10G064500 Glyma.07G063100 Glyma.08G113700 Glyma.09G232600 Glyma.06G068200 Glyma.01G199700 Glyma.12G181100 Glyma.08G007200 Glyma.05G154600 Glyma.03G029900 Glyma.08G321100 Glyma.16G044900 Glyma.02G241000 Glyma.15G206800 Glyma.U013500 Glyma.15G112500 Glyma.08G200100 Glyma.16G136900 Glyma.20G132300 Glyma.03G025600 Glyma.20G174800 Glyma.03G213300 Glyma.09G185500 Glyma.19G076500 Glyma.01G219400 Glyma.04G137200 Glyma.06G319700 Glyma.10G228900 Glyma.06G302400 Glyma.08G118500
QH34CK1A
QH34CK2A
QH34CK3A
DN50CK1A
DN50CK2A
DN50CK3A
QH34021A
QH34022A
QH34023A
QH34041A
QH34042A
QH34043A
QH34081A
QH34082A
QH34083A
DN50021A
DN50022A
DN50023A
DN50041A
DN50042A
DN50043A
DN50081A
DN50082A
DN50083A
Fig. S4 Heatmap of oxidoreductase activity signaling pathway. CK, 02, 04 and 08 represent 0, 2, 4 and 8 hours of salt treatment, respectively. 1A, 2A and 3A represent three biological replicates.
