# Supplementary material for: Time-series transcriptome comparison reveals the gene regulation network under salt stress in soybean (Glycine max) roots
Source: BMC Plant Biol. 2022 Mar 31;22:157. doi: 10.1186/s12870-022-03541-9 (PMC8969339; doi:10.1186/s12870-022-03541-9)
Supplement: Supplementary file 5 — Additional file 5: Fig. S5. Heatmap of negative regulation of proteolysis signaling pathway. [file 12870_2022_3541_MOESM5_ESM.pptx]

## Slide 1
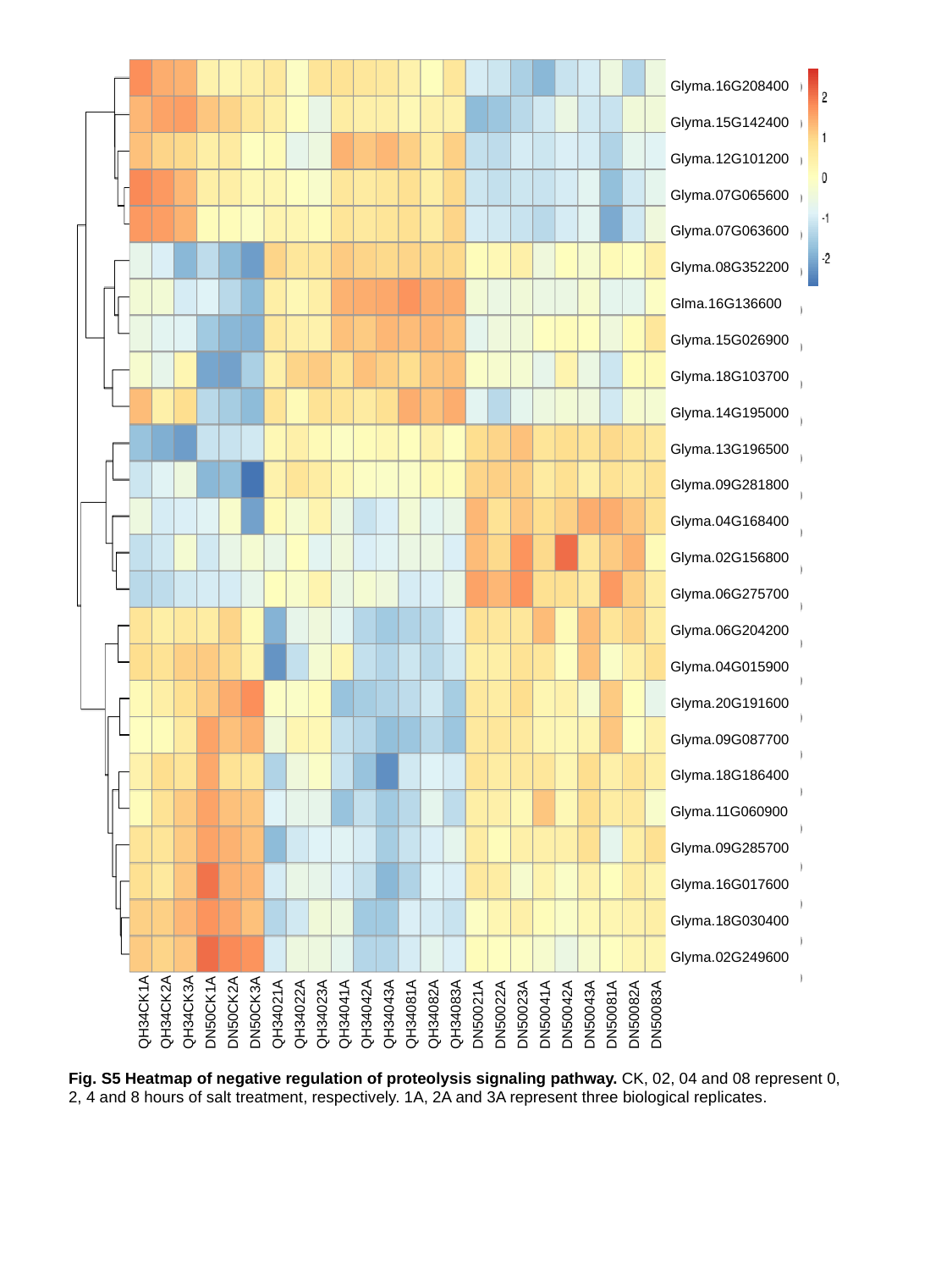

Glyma.16G208400 Glyma.15G142400 Glyma.12G101200 Glyma.07G065600 Glyma.07G063600 Glyma.08G352200 Glma.16G136600 Glyma.15G026900 Glyma.18G103700 Glyma.14G195000 Glyma.13G196500 Glyma.09G281800 Glyma.04G168400 Glyma.02G156800 Glyma.06G275700 Glyma.06G204200 Glyma.04G015900 Glyma.20G191600 Glyma.09G087700 Glyma.18G186400 Glyma.11G060900 Glyma.09G285700 Glyma.16G017600 Glyma.18G030400 Glyma.02G249600
QH34CK1A
QH34CK2A
QH34CK3A
DN50CK1A
DN50CK2A
DN50CK3A
QH34021A
QH34022A
QH34023A
QH34041A
QH34042A
QH34043A
QH34081A
QH34082A
QH34083A
DN50021A
DN50022A
DN50023A
DN50041A
DN50042A
DN50043A
DN50081A
DN50082A
DN50083A
Fig. S5 Heatmap of negative regulation of proteolysis signaling pathway. CK, 02, 04 and 08 represent 0, 2, 4 and 8 hours of salt treatment, respectively. 1A, 2A and 3A represent three biological replicates.
