# Supplementary material for: Time-series transcriptome comparison reveals the gene regulation network under salt stress in soybean (Glycine max) roots
Source: BMC Plant Biol. 2022 Mar 31;22:157. doi: 10.1186/s12870-022-03541-9 (PMC8969339; doi:10.1186/s12870-022-03541-9)
Supplement: Supplementary file 6 — Additional file 6: Fig. S6. GO enrichment analysis for uDEGs. [file 12870_2022_3541_MOESM6_ESM.pptx]

## Slide 1
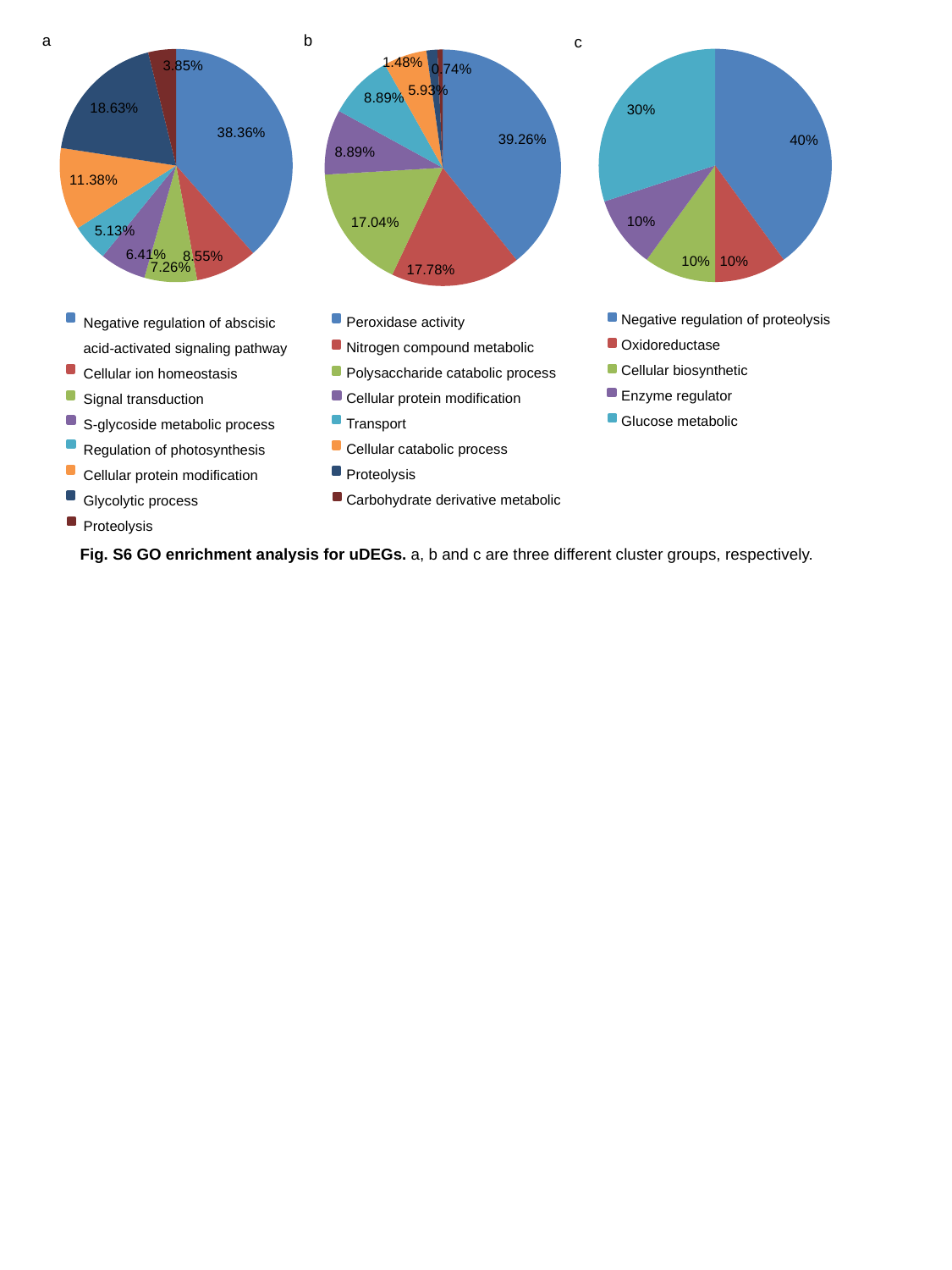

### Chart
| Category | |
|---|---|
| Negative regulation of proteolysis | 0.4 |
| Oxidoreductase | 0.1 |
| Clellular biosynthetic | 0.1 |
| Enzyme regulator | 0.1 |
| Glucose metabolic | 0.3 |
### Chart
| Category | |
|---|---|
| Peroxidase activity | 0.3926 |
| Nitrogen compound metabolic | 0.1778 |
| Polysaccharide catabolic process | 0.1704 |
| Cellular protein modification | 0.0889 |
| Transport | 0.0889 |
| Cellular catabolic process | 0.0593 |
| Proteolysis | 0.0148 |
| Carbohydrate derivative metabolic | 0.0074 |
### Chart
| Category | |
|---|---|
| Negative regulation of abscisic acid-activated signaling pathway | 0.3836 |
| Cellular ion homeostasis | 0.0855 |
| Signal transduction | 0.0726 |
| S-glycoside metabolic process | 0.0641 |
| Regulation of photosynthesis | 0.0513 |
| Cellular protein modification | 0.1138 |
| Glycolytic process | 0.1863 |
| Proteolysis | 0.0385 |a
b
c
Negative regulation of proteolysis
Oxidoreductase
Cellular biosynthetic
Enzyme regulator
Glucose metabolic
Peroxidase activity
Nitrogen compound metabolic
Polysaccharide catabolic process
Cellular protein modification
Transport
Cellular catabolic process
Proteolysis
Carbohydrate derivative metabolic
Negative regulation of abscisic acid-activated signaling pathway
Cellular ion homeostasis
Signal transduction
S-glycoside metabolic process
Regulation of photosynthesis
Cellular protein modification
Glycolytic process
Proteolysis
Fig. S6 GO enrichment analysis for uDEGs. a, b and c are three different cluster groups, respectively.
