# Supplementary material for: Time-series transcriptome comparison reveals the gene regulation network under salt stress in soybean (Glycine max) roots
Source: BMC Plant Biol. 2022 Mar 31;22:157. doi: 10.1186/s12870-022-03541-9 (PMC8969339; doi:10.1186/s12870-022-03541-9)
Supplement: Supplementary file 7 — Additional file 7: Fig. S7. KEGG pathway analysis of uDEGs. [file 12870_2022_3541_MOESM7_ESM.pptx]

## Slide 1
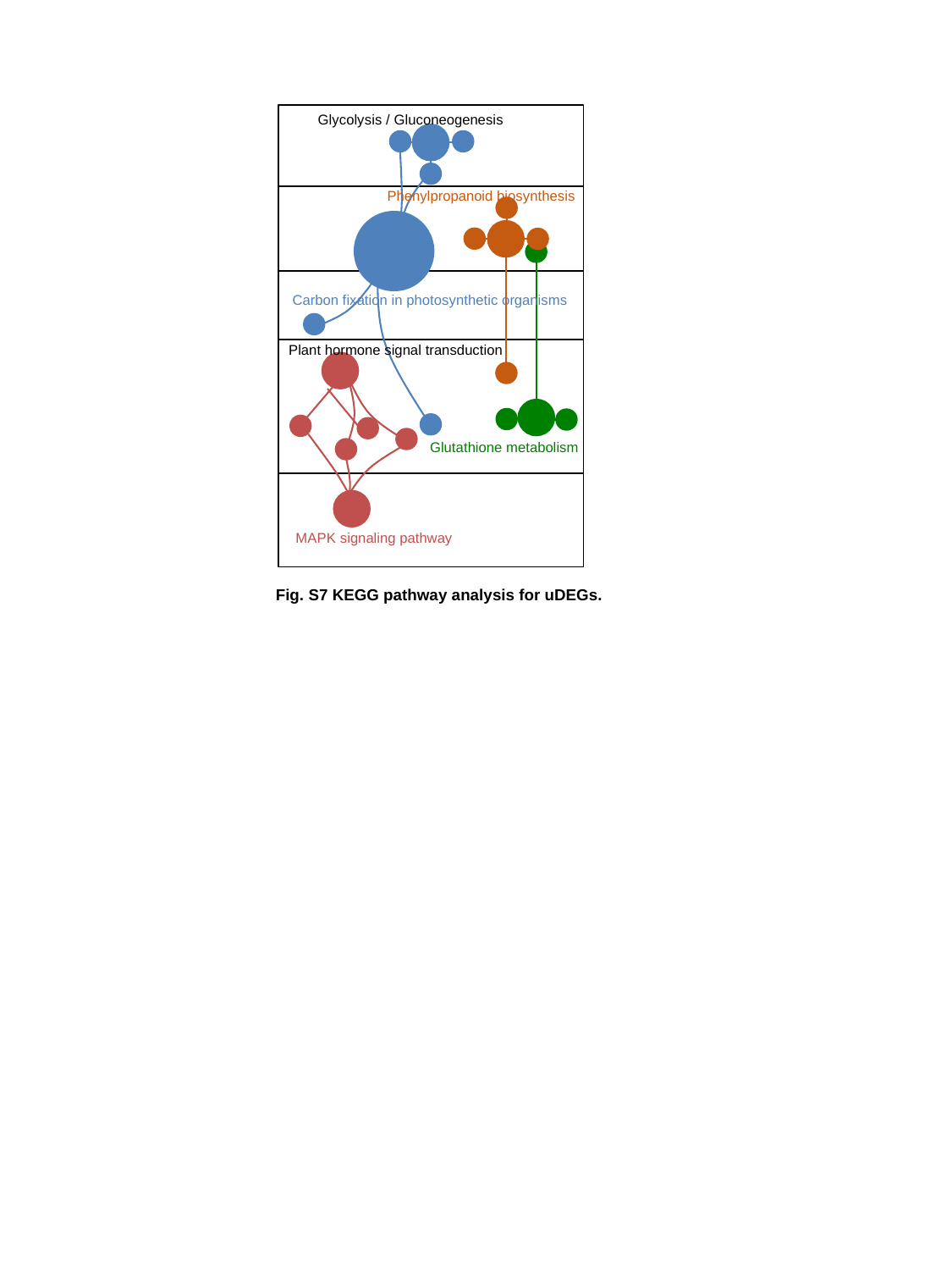

Glycolysis / Gluconeogenesis
Phenylpropanoid biosynthesis
Carbon fixation in photosynthetic organisms
Plant hormone signal transduction
Glutathione metabolism
MAPK signaling pathway
Fig. S7 KEGG pathway analysis for uDEGs.
