# Supplementary material for: Time-series transcriptome comparison reveals the gene regulation network under salt stress in soybean (Glycine max) roots
Source: BMC Plant Biol. 2022 Mar 31;22:157. doi: 10.1186/s12870-022-03541-9 (PMC8969339; doi:10.1186/s12870-022-03541-9)
Supplement: Supplementary file 8 — Additional file 8: Fig. S8. Pathway analysis of gene network clusters for sDEGs. [file 12870_2022_3541_MOESM8_ESM.pptx]

## Slide 1
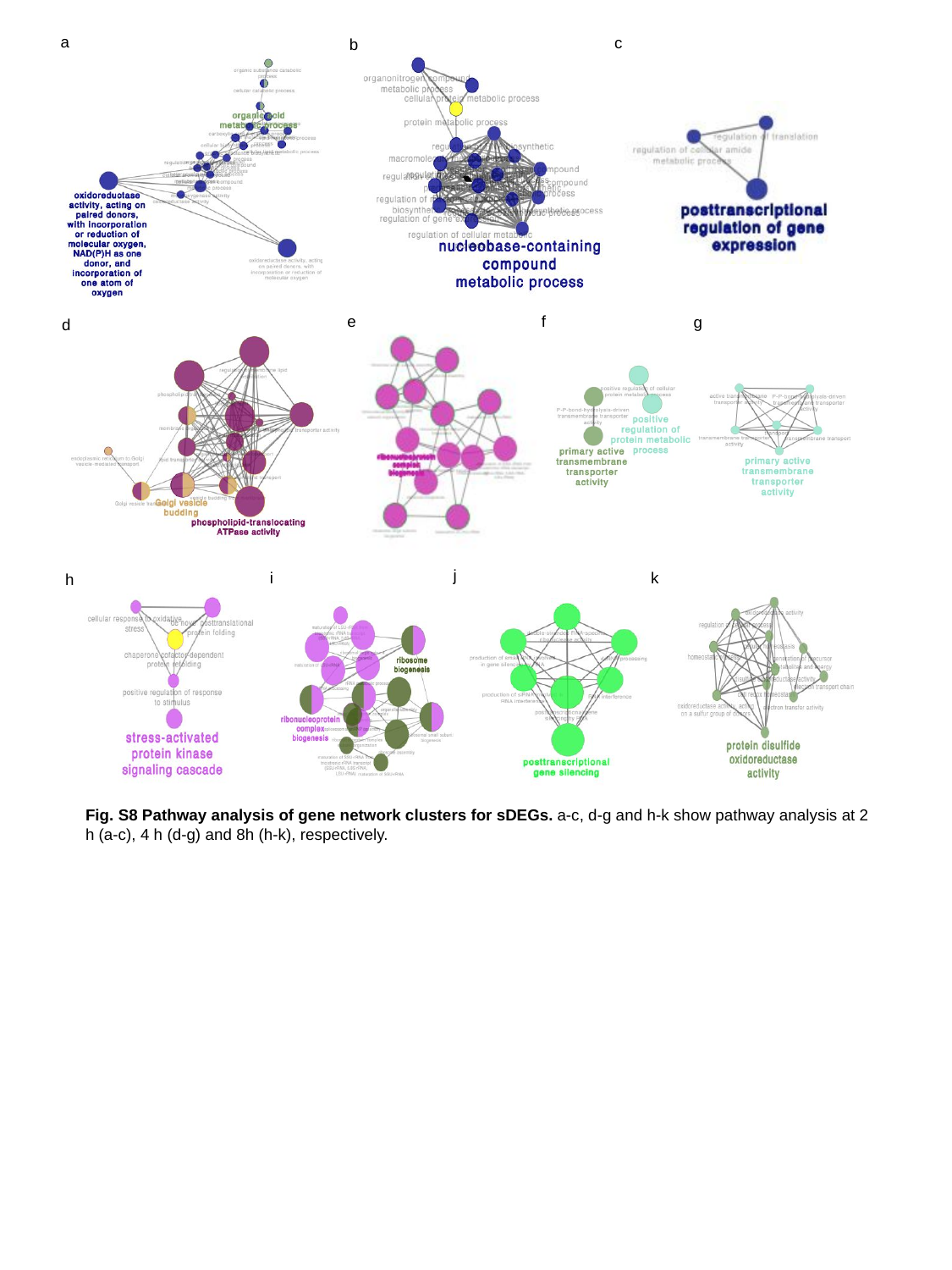

a
c
b
e
f
g
d
j
k
i
h
Fig. S8 Pathway analysis of gene network clusters for sDEGs. a-c, d-g and h-k show pathway analysis at 2 h (a-c), 4 h (d-g) and 8h (h-k), respectively.
