# Supplementary material for: Time-series transcriptome comparison reveals the gene regulation network under salt stress in soybean (Glycine max) roots
Source: BMC Plant Biol. 2022 Mar 31;22:157. doi: 10.1186/s12870-022-03541-9 (PMC8969339; doi:10.1186/s12870-022-03541-9)
Supplement: Supplementary file 9 — Additional file 9: Fig. S9. Heatmap of hormone signaling pathways. [file 12870_2022_3541_MOESM9_ESM.pptx]

## Slide 1
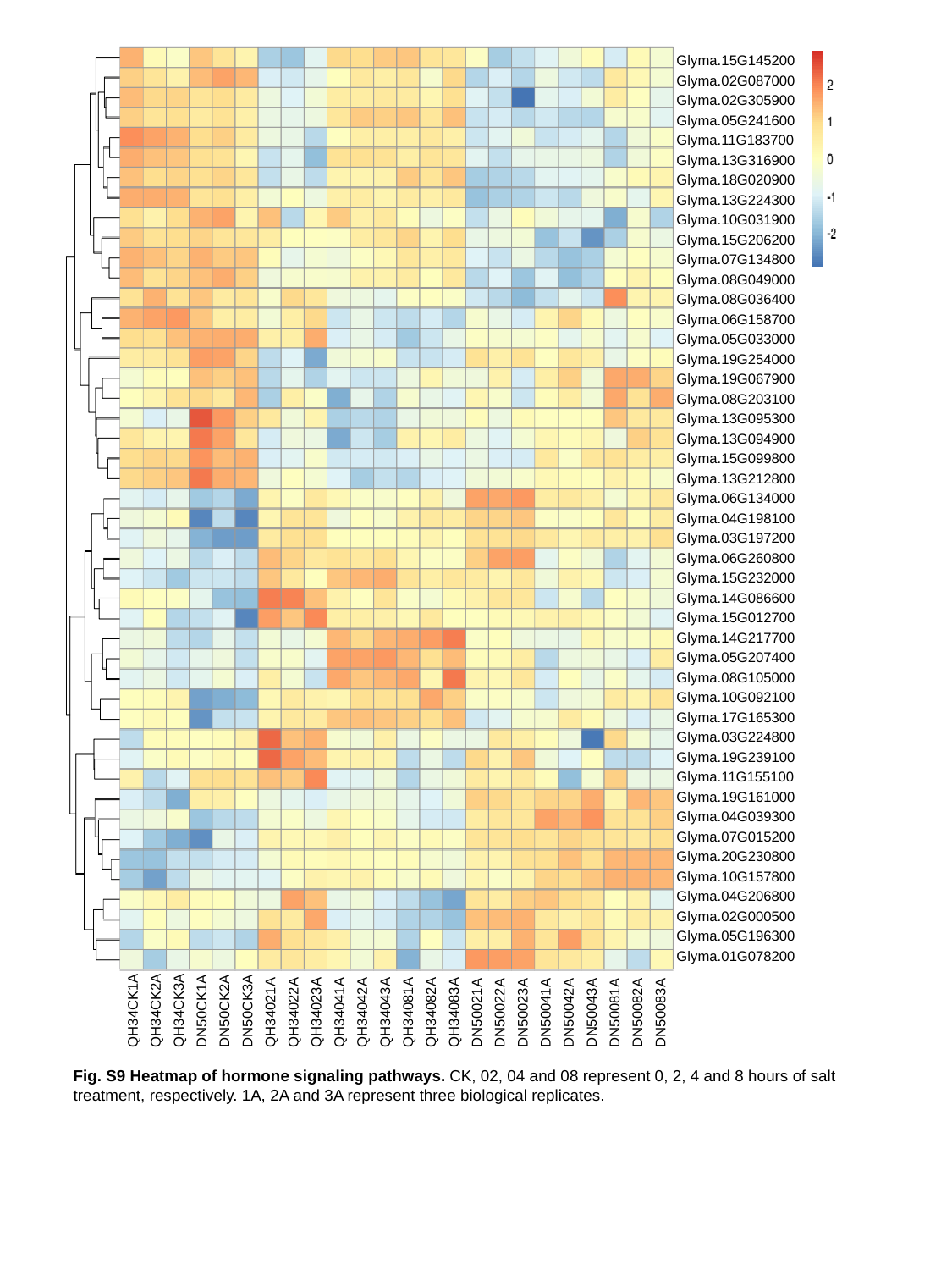

Glyma.15G145200 Glyma.02G087000 Glyma.02G305900 Glyma.05G241600 Glyma.11G183700 Glyma.13G316900 Glyma.18G020900 Glyma.13G224300 Glyma.10G031900 Glyma.15G206200 Glyma.07G134800 Glyma.08G049000 Glyma.08G036400 Glyma.06G158700 Glyma.05G033000 Glyma.19G254000 Glyma.19G067900 Glyma.08G203100 Glyma.13G095300 Glyma.13G094900 Glyma.15G099800 Glyma.13G212800 Glyma.06G134000 Glyma.04G198100 Glyma.03G197200 Glyma.06G260800 Glyma.15G232000 Glyma.14G086600 Glyma.15G012700 Glyma.14G217700 Glyma.05G207400 Glyma.08G105000 Glyma.10G092100 Glyma.17G165300 Glyma.03G224800 Glyma.19G239100 Glyma.11G155100 Glyma.19G161000 Glyma.04G039300 Glyma.07G015200 Glyma.20G230800 Glyma.10G157800 Glyma.04G206800 Glyma.02G000500 Glyma.05G196300 Glyma.01G078200
QH34CK1A
QH34CK2A
QH34CK3A
DN50CK1A
DN50CK2A
DN50CK3A
QH34021A
QH34022A
QH34023A
QH34041A
QH34042A
QH34043A
QH34081A
QH34082A
QH34083A
DN50021A
DN50022A
DN50023A
DN50041A
DN50042A
DN50043A
DN50081A
DN50082A
DN50083A
Fig. S9 Heatmap of hormone signaling pathways. CK, 02, 04 and 08 represent 0, 2, 4 and 8 hours of salt treatment, respectively. 1A, 2A and 3A represent three biological replicates.
