# Supplementary material for: Time-series transcriptome comparison reveals the gene regulation network under salt stress in soybean (Glycine max) roots
Source: BMC Plant Biol. 2022 Mar 31;22:157. doi: 10.1186/s12870-022-03541-9 (PMC8969339; doi:10.1186/s12870-022-03541-9)
Supplement: Supplementary file 10 — Additional file 10: Fig. S10. Heatmap of MAPK signaling pathway. [file 12870_2022_3541_MOESM10_ESM.pptx]

## Slide 1
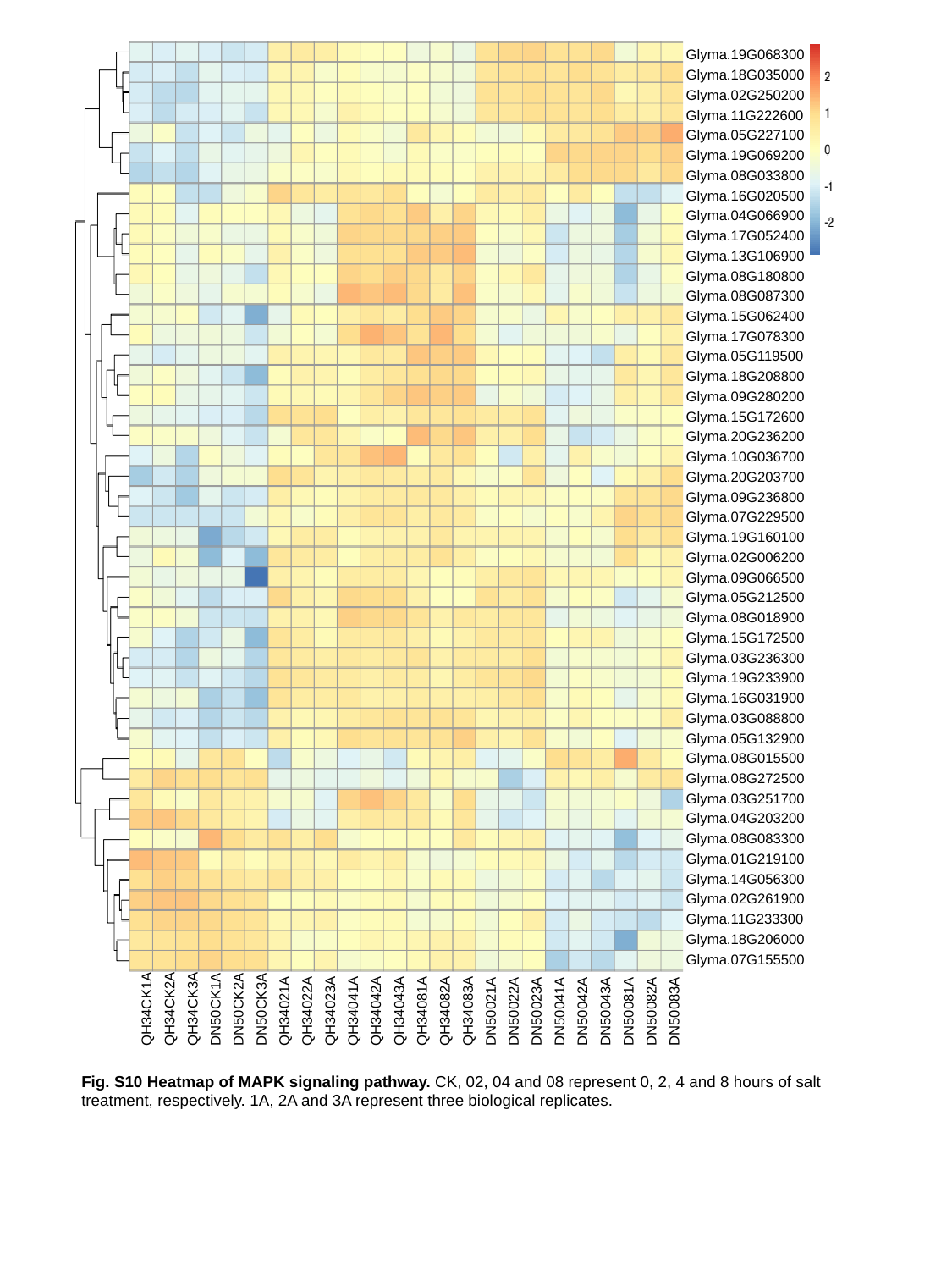

Glyma.19G068300 Glyma.18G035000 Glyma.02G250200 Glyma.11G222600 Glyma.05G227100 Glyma.19G069200 Glyma.08G033800 Glyma.16G020500 Glyma.04G066900 Glyma.17G052400 Glyma.13G106900 Glyma.08G180800 Glyma.08G087300 Glyma.15G062400 Glyma.17G078300 Glyma.05G119500 Glyma.18G208800 Glyma.09G280200 Glyma.15G172600 Glyma.20G236200 Glyma.10G036700 Glyma.20G203700 Glyma.09G236800 Glyma.07G229500 Glyma.19G160100 Glyma.02G006200 Glyma.09G066500 Glyma.05G212500 Glyma.08G018900 Glyma.15G172500 Glyma.03G236300 Glyma.19G233900 Glyma.16G031900 Glyma.03G088800 Glyma.05G132900 Glyma.08G015500 Glyma.08G272500 Glyma.03G251700 Glyma.04G203200 Glyma.08G083300 Glyma.01G219100 Glyma.14G056300 Glyma.02G261900 Glyma.11G233300 Glyma.18G206000 Glyma.07G155500
QH34CK1A
QH34CK2A
QH34CK3A
DN50CK1A
DN50CK2A
DN50CK3A
QH34021A
QH34022A
QH34023A
QH34041A
QH34042A
QH34043A
QH34081A
QH34082A
QH34083A
DN50021A
DN50022A
DN50023A
DN50041A
DN50042A
DN50043A
DN50081A
DN50082A
DN50083A
Fig. S10 Heatmap of MAPK signaling pathway. CK, 02, 04 and 08 represent 0, 2, 4 and 8 hours of salt treatment, respectively. 1A, 2A and 3A represent three biological replicates.
