# Supplementary material for: Time-series transcriptome comparison reveals the gene regulation network under salt stress in soybean (Glycine max) roots
Source: BMC Plant Biol. 2022 Mar 31;22:157. doi: 10.1186/s12870-022-03541-9 (PMC8969339; doi:10.1186/s12870-022-03541-9)
Supplement: Supplementary file 11 — Additional file 11: Fig. S11. Heatmap of phenylpropanoid biosynthesis signaling pathway. [file 12870_2022_3541_MOESM11_ESM.pptx]

## Slide 1
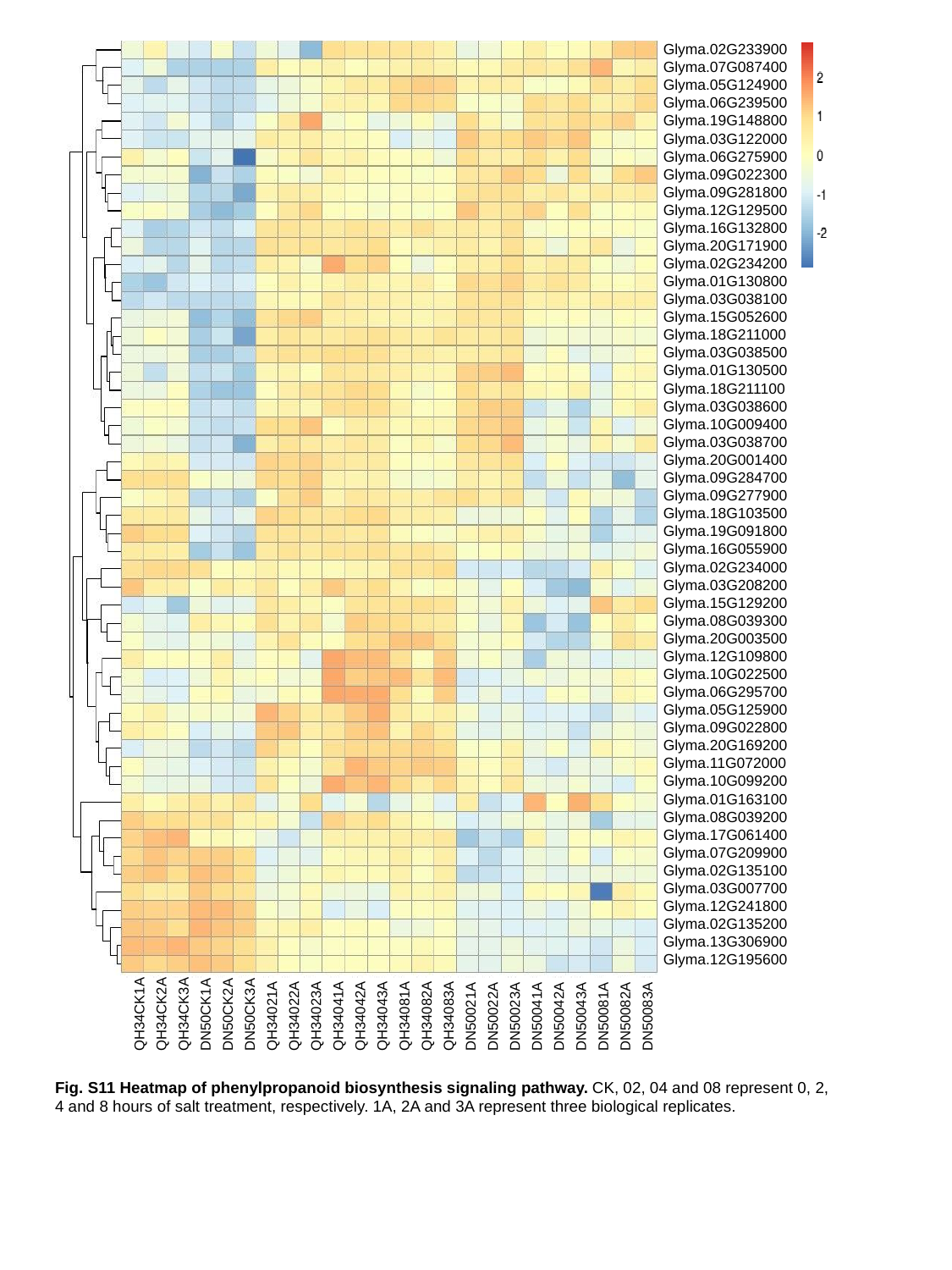

Glyma.02G233900 Glyma.07G087400 Glyma.05G124900 Glyma.06G239500 Glyma.19G148800 Glyma.03G122000 Glyma.06G275900 Glyma.09G022300 Glyma.09G281800 Glyma.12G129500 Glyma.16G132800 Glyma.20G171900 Glyma.02G234200 Glyma.01G130800 Glyma.03G038100 Glyma.15G052600 Glyma.18G211000 Glyma.03G038500 Glyma.01G130500 Glyma.18G211100 Glyma.03G038600 Glyma.10G009400 Glyma.03G038700 Glyma.20G001400 Glyma.09G284700 Glyma.09G277900 Glyma.18G103500 Glyma.19G091800 Glyma.16G055900 Glyma.02G234000 Glyma.03G208200 Glyma.15G129200 Glyma.08G039300 Glyma.20G003500 Glyma.12G109800 Glyma.10G022500 Glyma.06G295700 Glyma.05G125900 Glyma.09G022800 Glyma.20G169200 Glyma.11G072000 Glyma.10G099200 Glyma.01G163100 Glyma.08G039200 Glyma.17G061400 Glyma.07G209900 Glyma.02G135100 Glyma.03G007700 Glyma.12G241800 Glyma.02G135200 Glyma.13G306900 Glyma.12G195600
QH34CK1A
QH34CK2A
QH34CK3A
DN50CK1A
DN50CK2A
DN50CK3A
QH34021A
QH34022A
QH34023A
QH34041A
QH34042A
QH34043A
QH34081A
QH34082A
QH34083A
DN50021A
DN50022A
DN50023A
DN50041A
DN50042A
DN50043A
DN50081A
DN50082A
DN50083A
Fig. S11 Heatmap of phenylpropanoid biosynthesis signaling pathway. CK, 02, 04 and 08 represent 0, 2, 4 and 8 hours of salt treatment, respectively. 1A, 2A and 3A represent three biological replicates.
