# Supplementary material for: Time-series transcriptome comparison reveals the gene regulation network under salt stress in soybean (Glycine max) roots
Source: BMC Plant Biol. 2022 Mar 31;22:157. doi: 10.1186/s12870-022-03541-9 (PMC8969339; doi:10.1186/s12870-022-03541-9)
Supplement: Supplementary file 12 — Additional file 12: Fig. S12. Heatmap of ribosome metabolism biosynthesis signaling pathway. [file 12870_2022_3541_MOESM12_ESM.pptx]

## Slide 1
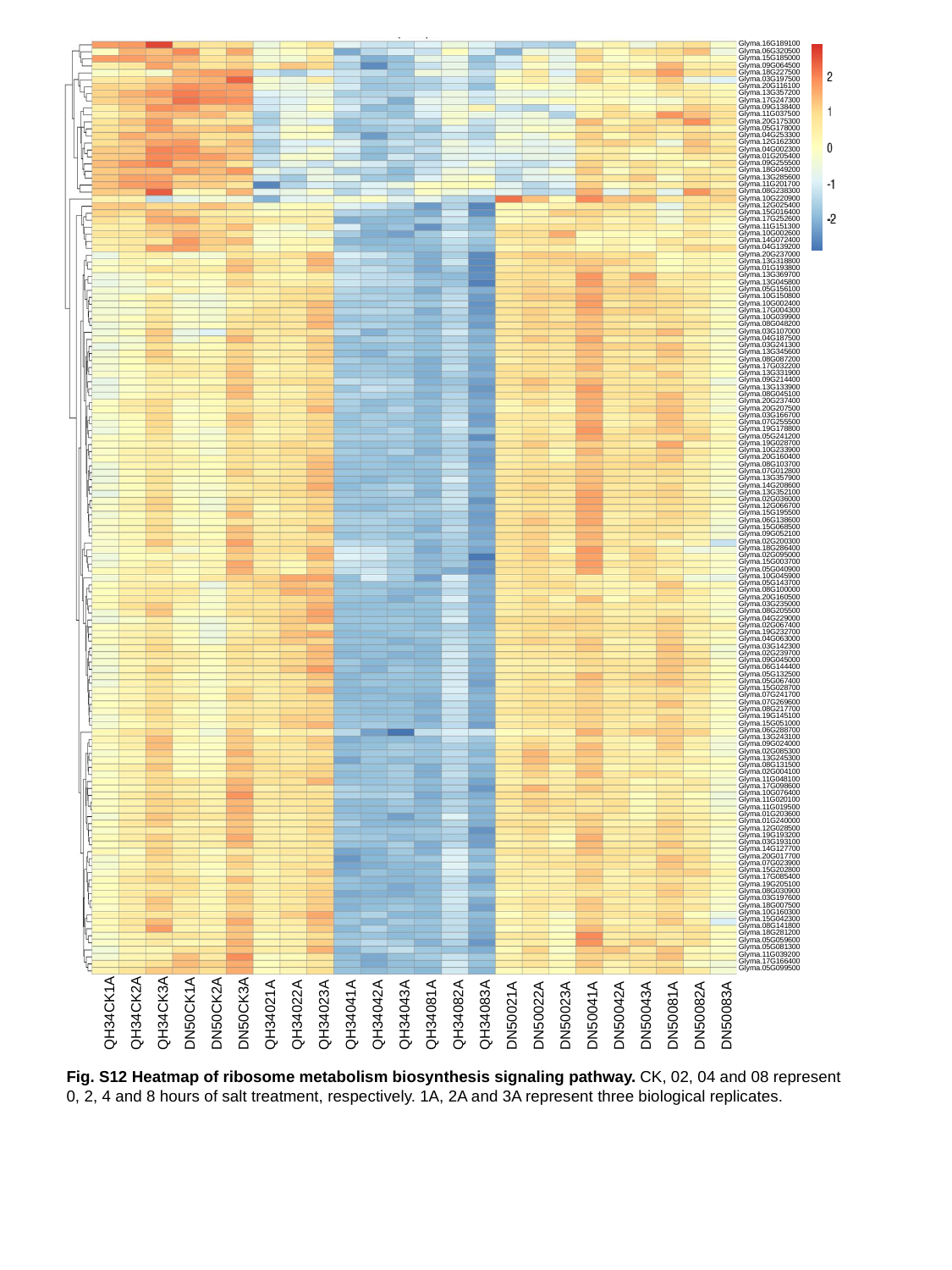

Glyma.16G189100 Glyma.06G320500 Glyma.15G185000 Glyma.09G064500 Glyma.18G227500 Glyma.03G197500 Glyma.20G116100 Glyma.13G357200 Glyma.17G247300 Glyma.09G138400 Glyma.11G037500 Glyma.20G175300 Glyma.05G178000 Glyma.04G253300 Glyma.12G162300 Glyma.04G002300 Glyma.01G205400 Glyma.09G255500 Glyma.18G049200 Glyma.13G285600 Glyma.11G201700 Glyma.08G238300 Glyma.10G220900 Glyma.12G025400 Glyma.15G016400 Glyma.17G252600 Glyma.11G151300 Glyma.10G002600 Glyma.14G072400 Glyma.04G139200 Glyma.20G237000 Glyma.13G318800 Glyma.01G193800 Glyma.13G369700 Glyma.13G045800 Glyma.05G156100 Glyma.10G150800 Glyma.10G002400 Glyma.17G004300 Glyma.10G039900 Glyma.08G048200 Glyma.03G107000 Glyma.04G187500 Glyma.03G241300 Glyma.13G345600 Glyma.08G087200 Glyma.17G032200 Glyma.13G331900 Glyma.09G214400 Glyma.13G133900 Glyma.08G045100 Glyma.20G237400 Glyma.20G207500 Glyma.03G166700 Glyma.07G255500 Glyma.19G178800 Glyma.05G241200 Glyma.19G028700 Glyma.10G233900 Glyma.20G160400 Glyma.08G103700 Glyma.07G012800 Glyma.13G357900 Glyma.14G208600 Glyma.13G352100 Glyma.02G036000 Glyma.12G066700 Glyma.15G195500 Glyma.06G138600 Glyma.15G068500 Glyma.09G052100 Glyma.02G200300 Glyma.18G286400 Glyma.02G095000 Glyma.15G003700 Glyma.05G040900 Glyma.10G045900 Glyma.05G143700 Glyma.08G100000 Glyma.20G160500 Glyma.03G235000 Glyma.08G205500 Glyma.04G229000 Glyma.02G067400 Glyma.19G232700 Glyma.04G063000 Glyma.03G142300 Glyma.02G239700 Glyma.09G045000 Glyma.06G144400 Glyma.05G132500 Glyma.05G067400 Glyma.15G028700 Glyma.07G241700 Glyma.07G269600 Glyma.08G217700 Glyma.19G145100 Glyma.15G051000 Glyma.06G288700 Glyma.13G243100 Glyma.09G024000 Glyma.02G085300 Glyma.13G245300 Glyma.08G131500 Glyma.02G004100 Glyma.11G048100 Glyma.17G098600 Glyma.10G076400 Glyma.11G020100 Glyma.11G019500 Glyma.01G203600 Glyma.01G240000 Glyma.12G028500 Glyma.19G193200 Glyma.03G193100 Glyma.14G127700 Glyma.20G017700 Glyma.07G023900 Glyma.15G202800 Glyma.17G085400 Glyma.19G205100 Glyma.08G030900 Glyma.03G197600 Glyma.18G007500 Glyma.10G160300 Glyma.15G042300 Glyma.08G141800 Glyma.18G281200 Glyma.05G059600 Glyma.05G081300 Glyma.11G039200 Glyma.17G166400 Glyma.05G099500
QH34CK1A
QH34CK2A
QH34CK3A
DN50CK1A
DN50CK2A
DN50CK3A
QH34021A
QH34022A
QH34023A
QH34041A
QH34042A
QH34043A
QH34081A
QH34082A
QH34083A
DN50021A
DN50022A
DN50023A
DN50041A
DN50042A
DN50043A
DN50081A
DN50082A
DN50083A
Fig. S12 Heatmap of ribosome metabolism biosynthesis signaling pathway. CK, 02, 04 and 08 represent 0, 2, 4 and 8 hours of salt treatment, respectively. 1A, 2A and 3A represent three biological replicates.
